# Southern rice black‐streaked dwarf virus hijacks SNARE complex of its insect vector for its effective transmission to rice

**DOI:** 10.1111/mpp.13109

**Published:** 2021-08-13

**Authors:** Lu Zhang, Wenwen Liu, Xiaowan Zhang, Li Li, Xifeng Wang

**Affiliations:** ^1^ State Key Laboratory for Biology of Plant Diseases and Insect Pests Institute of Plant Protection Chinese Academy of Agricultural Sciences Beijing China

**Keywords:** dissemination, insect vector, SNAREs, southern rice black‐streaked dwarf virus (SRBSDV), transmission, vesicle

## Abstract

Vesicular trafficking is an important dynamic process that facilitates intracellular transport of biological macromolecules and their release into the extracellular environment. However, little is known about whether or how plant viruses utilize intracellular vesicles to their advantage. Here, we report that southern rice black‐streaked dwarf virus (SRBSDV) enters intracellular vesicles in epithelial cells of its insect vector by engaging VAMP7 and Vti1a proteins in the soluble *N*‐ethylmaleimide‐sensitive factor attachment protein receptor (SNARE) complex. The major outer capsid protein P10 of SRBSDV was shown to interact with VAMP7 and Vti1a of the white‐backed planthopper and promote the fusion of vesicles into a large vesicle, which finally fused with the plasma membrane to release virions from midgut epithelial cells. Downregulation of the expression of either VAMP7 or Vti1a did not affect viral entry and accumulation in the gut, but significantly reduced viral accumulation in the haemolymph. It also did not affect virus acquisition, but significantly reduced the virus transmission efficiency to rice. Our data reveal a critical mechanism by which a plant reovirus hijacks the vesicle transport system to overcome the midgut escape barrier in vector insects and provide new insights into the role of the SNARE complex in viral transmission and the potential for developing novel strategies of viral disease control.

## Graphical abstract

The major outer capsid protein P10 of southern rice black‐streaked dwarf virus interacts with VAMP7 and Vti1a proteins in the SNARE complex in midgut epithelial cells of the white‐backed planthopper to promote vesicle fusion and virion release.

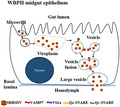

## 1 INTRODUCTION

Southern rice black‐streaked dwarf virus (SRBSDV, genus *Fijivirus*, family *Reoviridae*) was first discovered in 2001 in Guangdong Province, China (Zhang et al., 2008; Zhou et al., 2008). SRBSDV then rapidly spread throughout rice‐growing areas of southern, central, and eastern China, northern Vietnam, South Korea, Japan, and Thailand, becoming one of the most important rice pathogens in East and South‐east Asia (Dinh et al., 2012; Hoang et al., 2011; Matsukura et al., 2013; Zhou et al., 2008). After virus infection, rice plants develop darkened leaves, white waxy or black‐streaked swellings along stem veins, and severe stunting, which lead to serious rice losses (Zhou et al., 2010, 2013). In China in 2010, over 1,360,000 ha of rice plants were affected by SRBSDV, leading to 30%–50% yield losses, and over 700,000 ha of rice plants were poorly harvested (Lv et al., 2017). In the same year, more than 60,000 ha of rice fields were infected by SRBSDV, causing yield failure in 29 provinces of Vietnam (Zhou et al., 2013). In more recent years, the damage from SRBSDV has decreased due to precautionary and integrated management, but it is still serious in some areas, and the risk of a severe epidemic is still high.

In fields, SRBSDV is only spread to host plants by the insect vector white‐backed planthopper (WBPH, *Sogatella furcifera*), a long‐distance migratory pest (Jia, Chen, Mao, et al., 2012; Liu et al., 2010; Pu et al., 2012). Therefore, the severity of this disease commonly depends on the virus transmission efficiency, population numbers, and migratory dispersal of WBPHs (Li et al., 2012; Zhou et al., 2013). The insects usually overwinter in subtropical and tropical areas and can be carried by wind currents from the south to north in early spring (Matsukura et al., 2017; Tu et al., 2013). Thus, SRBSDV is carried by WBPHs to rice fields in new areas (Pu et al., 2012; Wang et al., 2010). Both adults and nymphs of WBPHs can transmit SRBSDV to rice with high efficiency (Pu et al., 2012). About 83% of newborn insects that feed on SRBSDV‐infected rice plants have been found to be viruliferous (Pu et al., 2012). A high proportion of viruliferous insects among the WBPH population can lead to secondary viral infections and serious outbreaks of the disease (Mar et al., 2014; Matsukura et al., 2017).

SRBSDV is effectively transmitted by WBPHs in a persistent propagative manner whereby the virus replicates and is retained by insects throughout their lives (Tu et al., 2013; Zhou et al., 2013). In the circulative propagative process, virions enter the alimentary canal and infect gut epithelial cells, replicate, cross the midgut release barrier to enter the haemolymph or other tissues, and then move into the salivary glands. Finally, the virus is transferred to a plant host from saliva during insect feeding (Hogenhout et al., 2008; Raccah, 2001). This process requires the virus to overcome multiple barriers in the host such as the midgut barrier, the immune response, and the salivary gland barrier (Bragard et al., 2013; Than et al., 2016; Ziegler & Brault, 2008). Some studies have shown that another planthopper species, small brown planthopper (SBPH, *Laodelphax striatellus*), can also acquire SRBSDV but cannot efficiently transmit the virus to plants. In this case, SRBSDV enters the epithelial cells of the SBPH midgut for replication, but fails to overcome the midgut release barrier into the haemocoel and does not enter the salivary glands (Jia, Chen, Mao, et al., 2012; Lan et al., 2015). Thus, the midgut barrier is crucial for SRBSDV transmission through insect vectors.

Commonly, reoviruses invade cells through receptor‐mediated, clathrin‐dependent endocytosis into the cytoplasm (Danthi et al., 2010; Ehrlich et al., 2004; Maginnis et al., 2008). The viruses replicate in their viroplasms, which contain viral double‐stranded (ds) RNAs and virus particles at various stages of maturation (Farsetta et al., 2000; Jia, Chen, Zheng, et al., 2012; Wei & Li, 2016). The newly mature virions are then released from the viroplasm via systemic dissemination, which requires the virus to effectively navigate diverse intracellular and extracellular environments (Boehme et al., 2009, 2011). Previous studies of cultured insect vector cells have shown that some plant reoviruses exploit virus‐induced tubules to enter neighbouring insect cells and spread from cell to cell (Liu et al., 2011; Wei et al., 2006). However, we found many SRBSDV virions in vesicles in epithelial cells, which might assist virion movement and release from the gut cells.

Targeted transport of cargo by vesicles between distinct organelles and to or from the cell surface is mediated by membrane fusion (Gao et al., 2017; Koseoglu et al., 2015; Yoon & Munson, 2018). Soluble *N*‐ethylmaleimide‐sensitive factor attachment protein receptors (SNAREs) are the minimal and essential machinery for mediating fusion in vesicular transport (Hesketh et al., 2014; Ward et al., 2000; Weber et al., 1998). A SNARE complex is made up of three Q‐SNAREs (Qa‐, Qb‐, and Qc) and one R‐SNARE, which are localized to different vesicle membranes and form a bridge to fuse the two membranes (D’Agostino et al., 2018; Kienle et al., 2009).

We previously used the major outer capsid protein P10 of SRBSDV as the bait to screen a cDNA library of WBPH and identified many P10‐interacting proteins, including two SNARE proteins: vesicle‐associated membrane protein 7 (VAMP7) and vesicle transport V‐SNARE protein (Vti1a) (Than et al., 2016). VAMP7 has been classified as an R‐SNARE and Vti1a as a Qb‐SNARE. They interact to enable vesicle fusion and thus mediate the transport of substances (Flowerdew & Burgoyne, 2009; Walter et al., 2014). In the present study, we found that newly assembled SRBSDV virions utilize vesicles for their transport in gut epithelial cells of insect vectors by binding VAMP7 and Vti1a. Our results clarify how a plant reovirus enters and exits insect vector cells by way of vesicles and reveal a new function of SNARE proteins in assisting the intracellular dissemination of a reovirus by vesicular trafficking.

## 2 RESULTS

### 2.1 SRBSDV virions are distributed in coated vesicles in midgut cells

**FIGURE 1:**
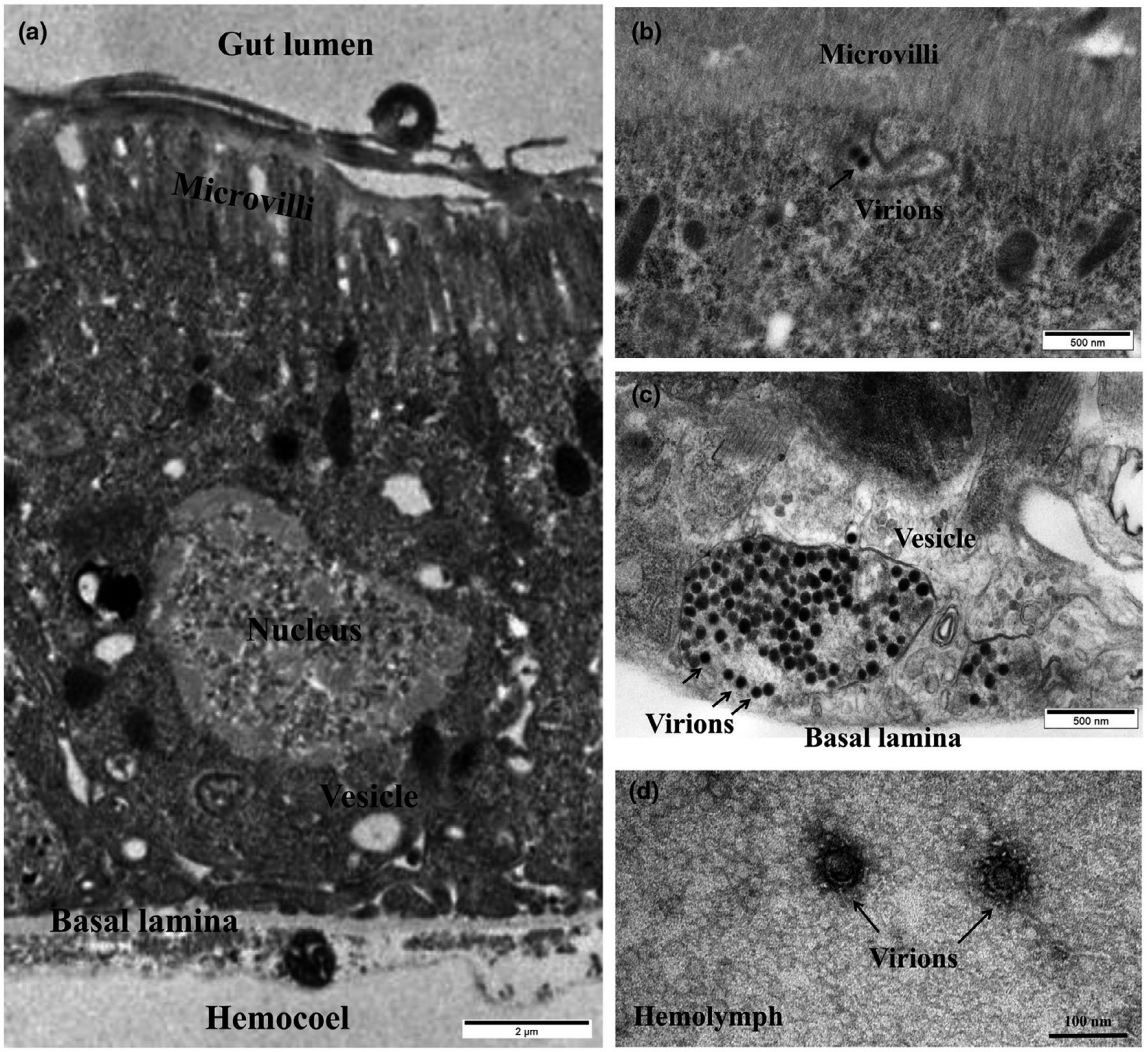
Transmission electron micrographs of SRBSDV virions in midgut epithelial cells of white‐backed planthoppers. (a) Midgut epithelial cell structure of the white‐backed planthopper. (b) Virions invaded intestinal epithelial cells via endocytosis. (c) Large vesicles were adjacent to the basal lamina when virions were being released from cells. (d) Virions in the haemolymph. Black arrow: SRBSDV virions

The insect midgut is the initial infection site and the first barrier to virus invasion (Hogenhout et al., 2008). The midgut of WBPH mainly consists of a single layer of epithelial cells, which include microvilli with an apical membrane on the lumen side and a basal membrane on the haemocoel side (Figure 1a). When the insects feed on fresh SRBSDV‐infected rice plants for 2 days, an acquisition efficiency of up to 80% can be achieved (Jia, Chen, Mao, et al., 2012; Pu et al., 2012). Thus, we chose a 2‐day virus acquisition access period (AAP). After allowing WBPHs a 2‐day AAP on SRBSDV‐infected rice plants, we reared the viruliferous WBPHs on healthy rice plants and excised the midguts to examine the distribution of SRBSDV virions in midgut epithelial cells using transmission electron microscopy (TEM). TEM analysis showed virions on the gut lumen side of the insect intestinal epithelial cells, which apparently entered through endocytosis (Figure 1b). However, the vesicles containing the virions were docked adjacent to the basal lamina of the midgut, and small vesicles with virions could be seen fusing with large vesicles (Figure 1c). In addition, TEM analysis showed that individual virions were not present in vesicles but scattered in the haemolymph (Figure 1d). Presumably, the virions could cross the insect's midgut via the fusion of vesicle and the basal lamina, followed by release into the haemolymph, and the large vesicles containing numerous virions might serve as a vehicle for more efficient dissemination of SRBSDV.

### 2.2 Multilateral interactions among SRBSDV P10, VAMP7, and Vti1a

**FIGURE 2:**
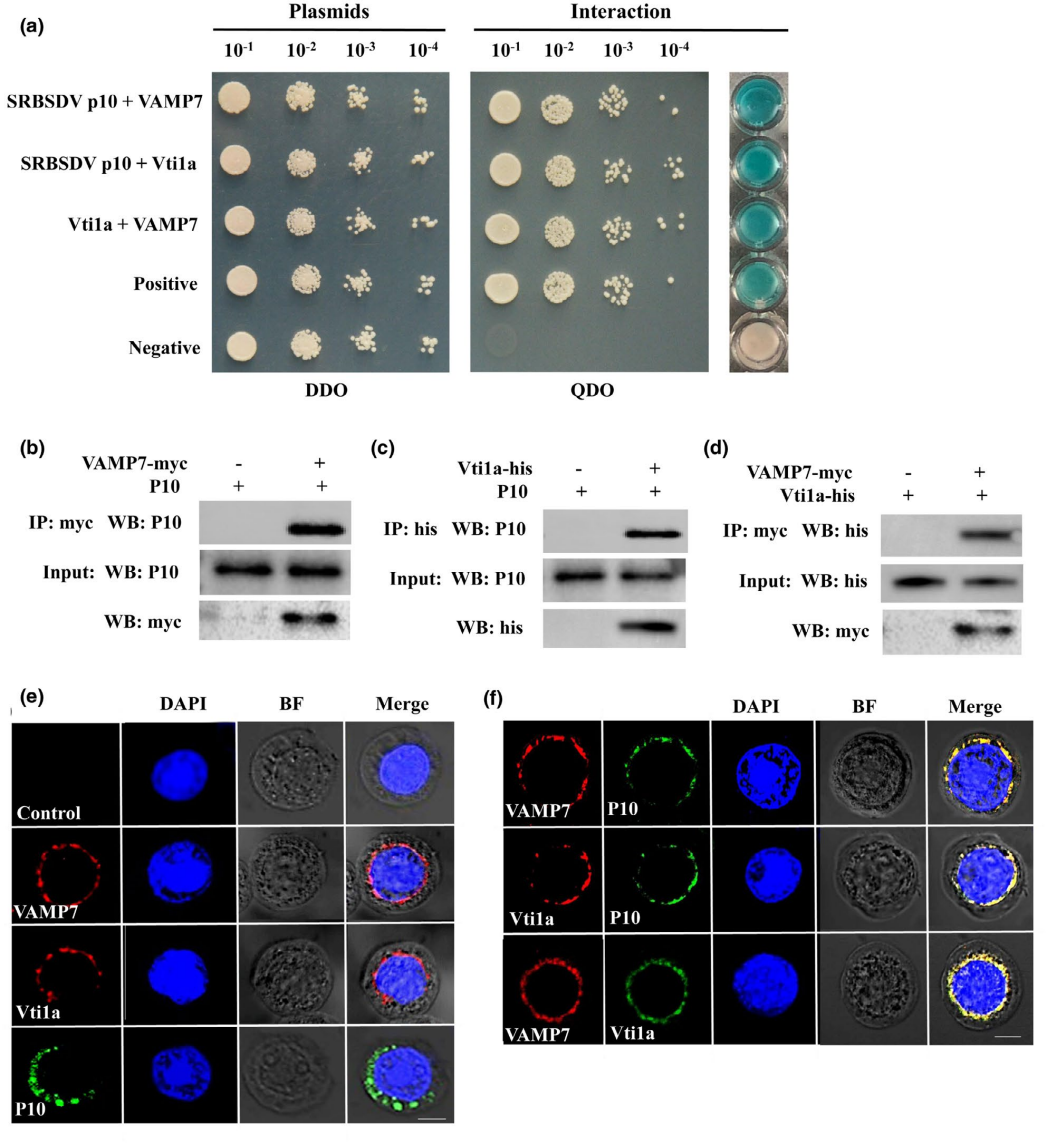
Interaction among SRBSDV capsid protein P10, VAMP7, and Vti1a in vivo and in vitro. (a) Interactions between SRBSDV P10 and VAMP7, SRBSDV P10 and Vti1a, and VAMP7 and Vti1a as detected using an in vivo yeast two‐hybrid assay. Yeast strain NMY51 was cotransformed with all possible pairs of the three proteins. Yeast cells, diluted 10^−1^ to 10^−4^ times, were plated onto DDO (SD−Trp−Leu) and QDO (SD−Trp−Leu−His−Ade) medium. Clones grown on DDO were selected for the β‐galactosidase assay. Large T + P53 was used as the positive control; large T + pPR3N served as the negative control. (b–d) Confirmation of protein interactions by in vitro coimmunoprecipitation. Sf9 cells were cotransfected with the respective recombinant bacmids (SRBSDV P10, VAMP7, and Vti1a) for protein expression. After cells were lysed in lysis buffer, the solution was incubated with anti‐bait antibody and protein A/G plus agarose beads for immunoprecipitation in each group. The anti‐prey antibody was used for western blot (WB) analysis to check for respective interactions. (e) Localization of the three proteins in Sf9 cells. The recombinant bacmids (SRBSDV P10, VAMP7, and Vti1a) were individually used to transfect Sf9 cells, which were observed with bright field (BF) and laser scanning confocal microscopy (LSCM). (f) Colocalization of three proteins in pairwise combinations. VAMP7 and P10 (top row), Vti1a and P10 (middle row), or VAMP7 and Vti1a (bottom) were coexpressed in Sf9 cells and observed with LSCM 72 hr after transfection. P10 was labelled with Dylight 488‐conjugated anti‐SRBSDV antibody (green), VAMP7 with Dylight 549‐conjugated anti‐VAMP7 antibody (red), and Vti1a with Dylight 488‐conjugated anti‐Vti1a antibody (green). Nuclei were stained with DAPI (blue). Noninfected Sf9 cells served as the negative control. Scale bars, 10 μm

To further explore the mechanism of SRBSDV dissemination via vesicles from midgut epithelium, we previously used SRBSDV P10 as the bait to screen a cDNA library of WBPH and identified two relevant vesicle membrane proteins, VAMP7 and Vti1a (Than et al., 2016). In the present study, we cloned the genes that encode VAMP7 (GenBank: MN764900) and Vti1a (GenBank: MN764901) from WBPH (Table S1). Their full open reading frames comprise 651 and 669 bp, respectively, encoding predicted proteins with 216 and 222 amino acids, each with one transmembrane domain but no signal peptide (Figure S1). We then used full‐length SRBSDV P10, VAMP7, and Vti1a in a yeast two‐hybrid (Y2H) assay to detect any interactions. The results showed that VAMP7 and Vti1a interacted strongly with SRBSDV P10 and also with each other (Figure 2a). To further confirm the interaction of these three proteins, we used Sf9 cells to express all pairwise combinations of SRBSDV P10, VAMP7, and Vti1a and tested the interactions using coimmunoprecipitation. We thus confirmed that SRBSDV P10, VAMP7, and Vti1a did interact with each other in all pairs (Figure 2b–d). We conducted additional Y2H studies to determine the interactions between SRBSDV P7‐1 and VAMP7 or Vti1a via cotransforming the bait plasmid PDHB1‐SRBSDV P7‐1(Mar et al., 2014) with pPR3N‐VAMP7 or pPR3N‐Vti1a through the Y2H system (Large T + P53 as positive control, pDHB1‐SRBSDV P7‐1 + pPR3N as negative control) and using a β‐galactosidase assay (Figure S2). The results indicated that there were no interactions between SRBSDV P7‐1 and VAMP7 or Vti1a, which suggests that SRBSDV P7‐1 does not participate in the vesicular trafficking mediated by VAMP7 or Vti1a.

For intracellular localization assays of SRBSDV P10, VAMP7, and Vti1a, Bac plasmids with the respective genes were each used alone to transfect cultured Sf9 cells or in pairs for cotransfection. After labelling with fluorescein‐conjugated antibodies, locations of proteins were observed via laser scanning confocal microscopy (LSCM). We observed that VAMP7 and Vti1a were distributed in the cytoplasm around the nucleus, but SRBSDV P10 was distributed throughout the cytoplasm (Figure 2e). In addition, VAMP7 colocalized with Vti1a in the cells (Figure 2f). Interestingly, when cells were cotransfected with SRBSDV P10 and either VAMP7 or Vti1a, SRBSDV P10 colocalized with the respective proteins in the cytoplasm surrounding the nucleus (Figure 2f). Thus, both VAMP7 and Vti1a interacted with SRBSDV P10 and seemed to alter the location of SRBSDV P10 in the cells.

### 2.3 The SNARE complex is involved in disseminating SRBSDV virions in the midgut epithelium

**FIGURE 3:**
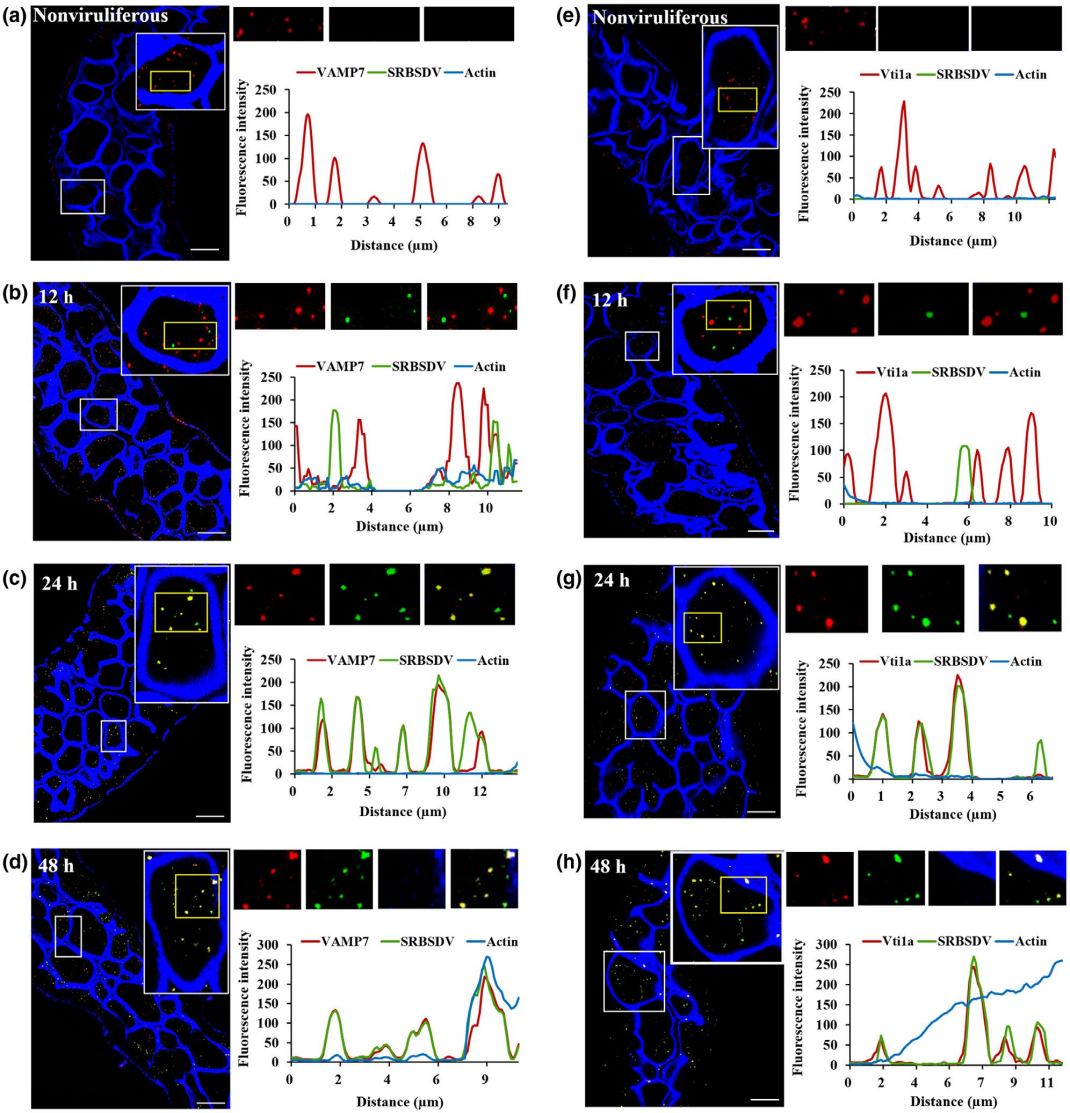
Colocalization of SRBSDV with VAMP7 and Vti1a in the midgut epithelium. Colocalization of SRBSDV with (a–d) VAMP7 or (e–h) Vti1a in white‐backed planthopper (WBPH) midgut cells was assessed using laser scanning confocal microscopy (LSCM) at different times after virus acquisition. Guts were incubated with anti‐SRBSDV antibody labelled with Dylight 488 (green) and anti‐VAMP7 or Vti1a antibody labelled with Dylight 549 (red) at 12, 24, and 48 hr after a 2‐day acquisition access period. Guts excised from nonviruliferous WBPHs were used as negative control. Samples were observed with LSCM. Scale bars, 20 µm. The fluorescence intensity was analysed using ImageJ

The midgut epithelium of WBPH was observed at different times after a 2‐day AAP using immunofluorescence and LSCM to localize the virions and either VAMP7 or Vti1a. Virions were labelled with Dylight 488‐conjugated anti‐SRBSDV (green), and VAMP7 or Vti1a was labelled with their corresponding antibodies conjugated to Dylight 549 (red). We observed many fluorescent puncta of VAMP7 or Vti1a in the midgut epithelial cells of nonviruliferous WBPHs (Figure 3a,e) and few virions in the midgut epithelial cells (Figures 3b,f and S3) at 12 hr after acquisition. In addition, very few virions colocalized with VAMP7 or Vti1a in the cytoplasm (Figures 3b,f and S3). However, after 24 hr, more virions were observed than after 12 hr, and they colocalized with VAMP7 and Vti1a, which were uniformly distributed throughout the cytoplasm (Figures 3c,g and S3). By 48 hr, virions were not only present in the cytoplasm, but also colocalized with VAMP7 and Vti1a on the cell membrane (Figures 3d,h and S3). These results suggest that both VAMP7 and Vti1a can bind SRBSDV in the midgut epithelial cells and are involved in disseminating virions in the midgut epithelium.

### 2.4 Interactions with VAMP7 and Vti1a assist virion entry into intracellular vesicles

**FIGURE 4:**
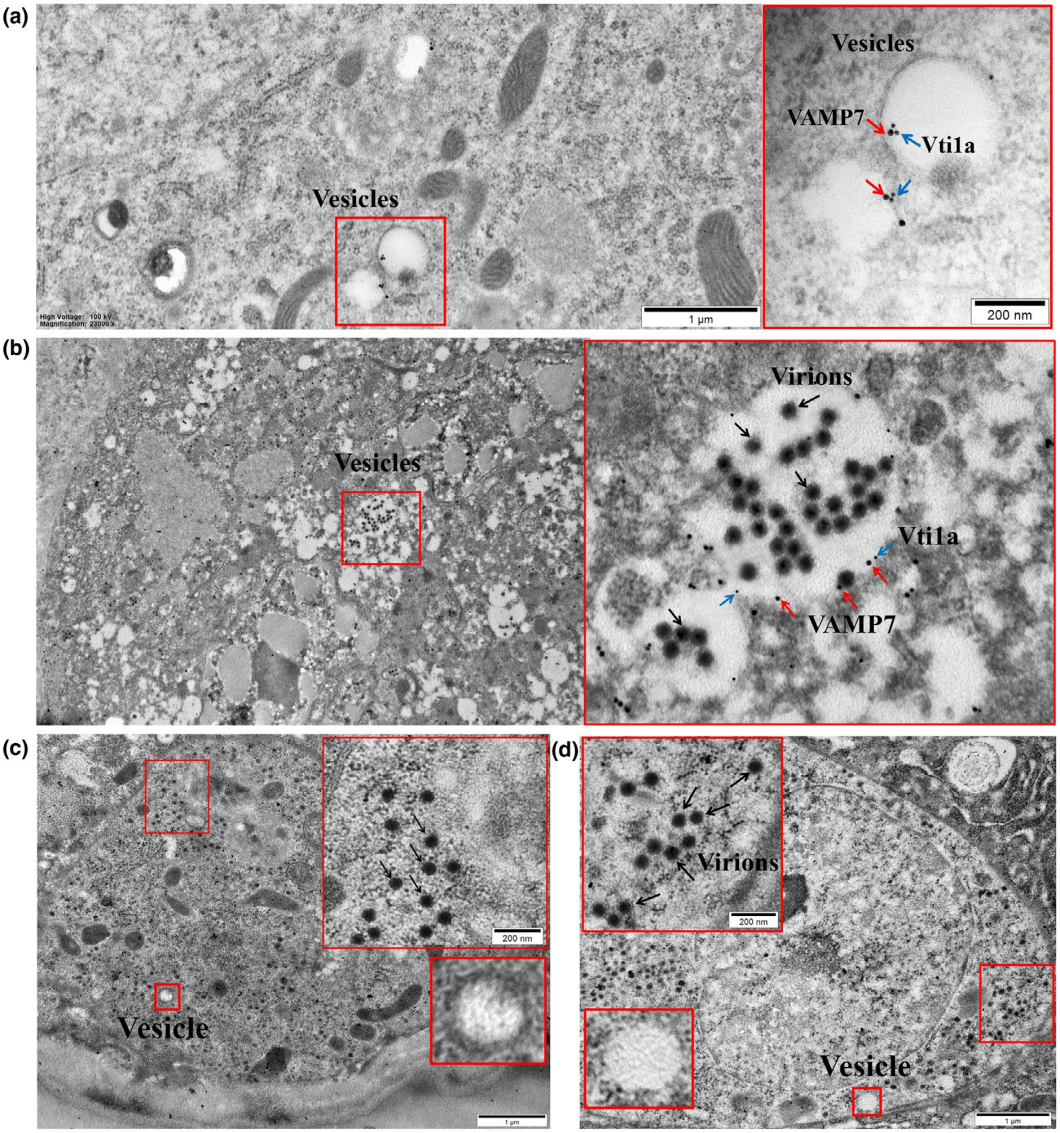
Downregulation of *VAMP7* or *Vti1a* expression inhibited entry of SRBSDV virions in vesicles. (a) Localization of VAMP7 and Vti1a in virus‐free epithelial cells. (b) SRBSDV, VAMP7, and Vti1a were distributed within virus‐infected epithelial cells of ds*GFP* control groups. (c, d) Distribution of SRBSDV in epithelial cells after downregulation of VAMP7 or Vti1a expression. Red boxes: partial enlarged view; red arrows: 10‐nm‐gold‐conjugated anti‐IgG against VAMP7; blue arrows: 5‐nm‐gold‐conjugated anti‐IgG against Vti1a; black arrows: SRBSDV virions

We injected dsRNA of *VAMP7* or *Vti1a* (ds*VAMP7* or ds*Vti1a*) into third‐instar nymphs of WBPH to interfere with expression of the two genes and then transferred the insects to healthy rice seedlings for 2 days. The injected nymphs were then moved to SRBSDV‐infected rice plants for a 2‐day AAP. The gut tissues of the nymphs were then excised and observed by TEM. Gut tissues from WBPHs that had fed for 2 days on an uninfected plant were used as a control. Based on our TEM observations, there were fewer fusion vesicles in cells without virions where VAMP7 and Vti1a were observed (Figure 4a). VAMP7 and Vti1a with colloidal gold localized together on the vesicle membrane in the control with no virions (Figure 4a). In addition, in gut epithelial cells of ds*GFP*‐injected insects that fed on SRBSDV‐infected plants, many gold particles of VAMP7 or Vti1a were also found on the membrane of fusing vesicles that contained numerous virions and some virions colocalized with VAMP7 or Vti1a on the membrane (Figure 4b). However, in the ds*VAMP7*‐ or ds*Vti1a*‐injected insects, numerous virions were also present in the cytoplasm of midgut epithelial cells but not in vesicles (Figure 4c,d). There were several small vesicles in the cytoplasm, but large vesicles were not observed in either ds*VAMP7*‐ or ds*Vti1a*‐treated insects (Figure 4c,d). These results suggested that SRBSDV virions cannot enter the intracellular vesicles in the absence of VAMP7 and Vti1a. They also indicated that VAMP7 and Vti1a may help the virions enter the vesicles and that the interaction among virions and these two proteins also promotes vesicle fusion.

### 2.5 Downregulation of either VAMP7 or Vti1a prevents the release of virions from epithelial cells, but does not affect viral replication

**FIGURE 5:**
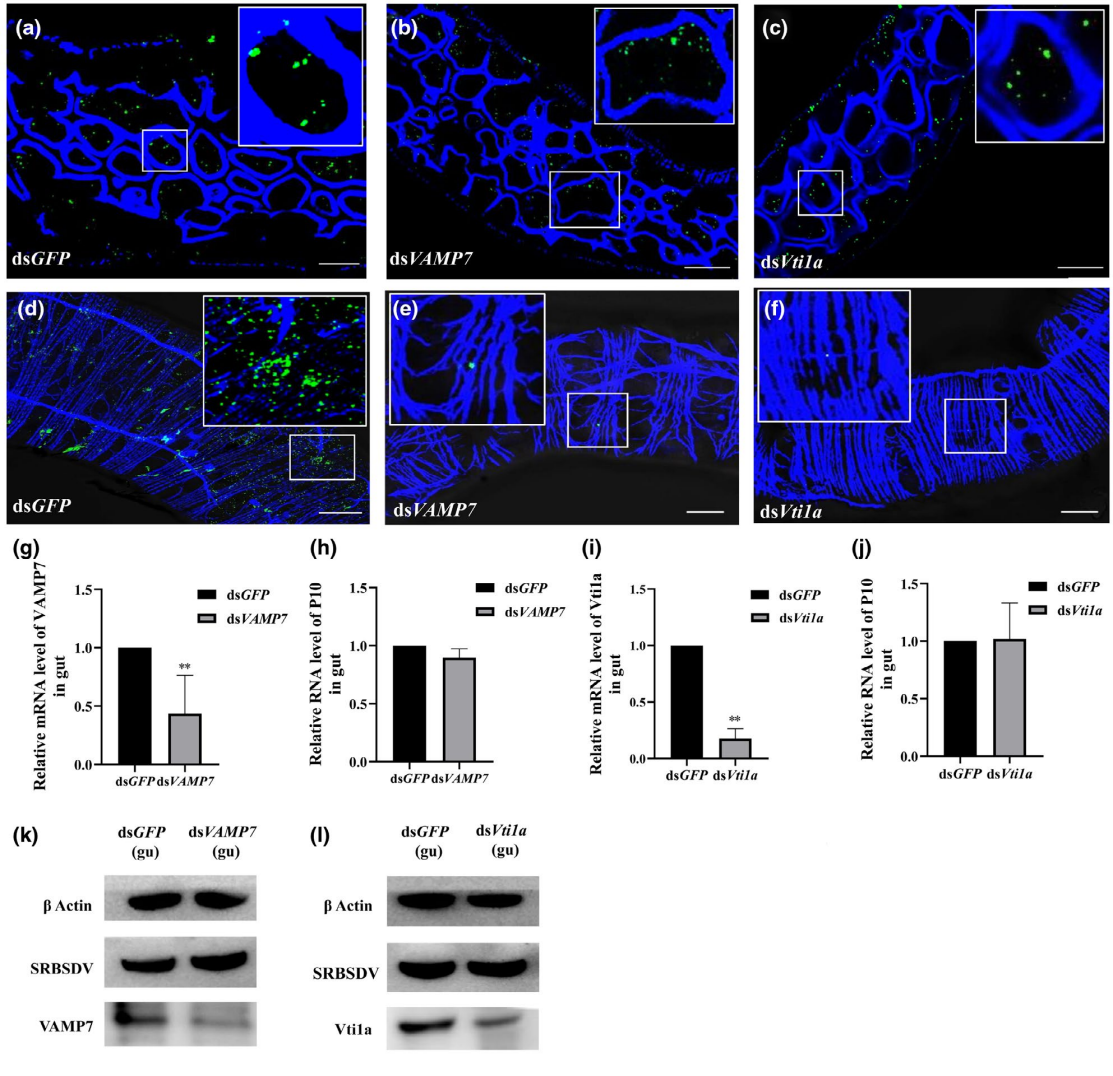
Downregulation of *VAMP7* or *Vti1a* did not affect viral entry or replication. (a–c) Localization of SRBSDV virions in the midgut epithelial cells from ds*GFP‐*, ds*VAMP7*‐, or ds*Vti1a‐*injected groups. Scale bars, 20 µm. (d–f) Localization of SRBSDV virions in the midgut muscle layer from ds*GFP*‐, ds*VAMP7*‐, or ds*Vti1a‐*injected nymphs. Guts were incubated with anti‐SRBSDV antibody conjugated with Dylight 488 (green). Scale bars, 20 µm. (g–j) The mRNA levels of *VAMP7* and *Vti1a* and the RNA levels of SRBSDV *P10* in guts of viruliferous white‐backed planthoppers were quantified using quantitative reverse transcription PCR in ds*GFP*‐, ds*VAMP7*‐, or ds*Vti1a*‐injected groups (mean ± *SEM* of three independent experiments, Student's *t* test, **p < 0.01). (k, l) Western blots to detect SRBSDV P10, VAMP7, and Vti1a in guts of viruliferous white‐backed planthoppers after injection with different dsRNAs

Nymphs were injected with ds*VAMP7*, ds*Vti1a*, or ds*GFP*. Then the dsRNA‐treated insects of these three groups all fed on the same SRBSDV‐infected leaf for 2 days to ensure that they were given access to the same amount of virus. When they finished the 2‐day AAP, they were transferred to healthy rice seedlings. The gut from each nymph was then dissected to examine cells for the presence of virions using LSCM. Many virions were present in the epithelial cells not only in the control groups but also in the ds*VAMP7*‐ and ds*Vti1a*‐injected groups at 48 hr after virus acquisition (Figure 5a–c). This result is consistent with TEM observations (Figure 4b–d). At 72 hr, we found numerous virions along the longitudinal muscle fibres of the gut in the control groups, but virions were rare in the ds*VAMP7*‐ and ds*Vti1a*‐injected groups (Figure 5d–f). Without the assistance of VAMP7 or Vti1a, very few virions were released from epithelial cells.

To verify whether VAMP7 and Vti1a are related to SRBSDV replication in the gut, we quantified the mRNA levels of *VAMP7*, *Vti1a*, and SRBSDV *P10* in the injected nymphs. The mRNA levels of *VAMP7* and *Vti1a* in the midgut of these nymphs were significantly lower than in the midgut of the ds*GFP*‐injected nymphs (Figure 5g,i). However, the RNA level of SRBSDV *P10* did not change significantly in the midgut in the ds*VAMP7*‐ and ds*Vti1a*‐injected nymphs (Figure 5h,j). The protein was also extracted from the guts of 100 nymphs after each treatment. We quantified the relative expression of SRBSDV P10, VAMP7, and Vti1a, using β‐actin as an internal reference. Compared with expression in the ds*GFP*‐injected group, the relative expression levels of VAMP7 and Vti1a were significantly lower, but those of SRBSDV P10 did not change significantly in either interference group (Figure 5k,l). These results suggested that downregulation of *VAMP7* or *Vti1a* had no significant influence on the SRBSDV titre in the guts of insects.

### 2.6 Downregulation of either VAMP7 or Vti1a prevents virion release from the gut to the haemolymph

**FIGURE 6:**
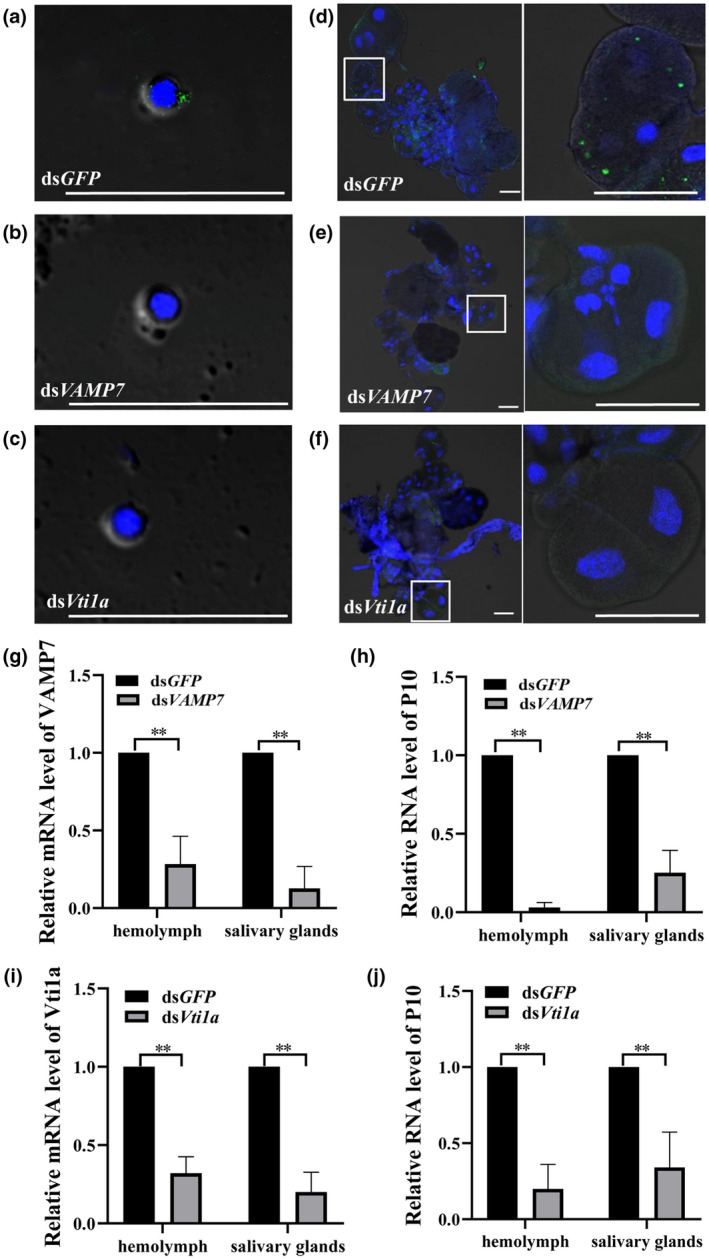
Downregulation of *VAMP7* or *Vti1a* prevented release of SRBSDV to haemolymph. (a–f) Haemocytes and salivary glands were incubated with anti‐SRBSDV antibody labelled with Dylight 488 (green) in different nymphs injected with ds*GFP*, ds*VAMP7*, or ds*Vti1a*. Scale bars, 50 µm. (g–j) The mRNA levels of *VAMP7* and *Vti1a* and RNA levels of SRBSDV *P10* in haemolymph and salivary glands of viruliferous white‐backed planthoppers injected with ds*GFP*‐, ds*VAMP7*‐, or ds*Vti1a* were quantified using quantitative reverse transcription PCR. Data are presented as the mean ± *SEM* of three independent experiments (Student's *t* test, **p < 0.01)

To explore the influence of the different treatments on dissemination in different tissues, we injected 300 third‐instar WBPH nymphs that had finished a 2‐day AAP with 23 nl of ds*GFP* (3 μg/μl), ds*VAMP7* (3 μg/μl), or ds*Vti1a* (3 μg/μl) and reared them on healthy rice seedlings. We isolated the haemolymph to look for virions in the ds*VAMP7*‐ and ds*Vti1a*‐injected nymphs. Some virions were present in the haemocytes from the ds*GFP*‐treated insects, but not in the ds*VAMP7*‐ or ds*Vti1a*‐injected insects at 6 days after virus acquisition (Figure 6a–c). SRBSDV virions were also found in the salivary glands from the control nymphs (Figure 6d); however, virions were observed in few salivary glands in the ds*VAMP7*‐ or ds*Vti1a*‐injected nymphs at 8 days after virus acquisition (Figures 6e,f and S4). Efficiency of the salivary glands of viruliferous WBPHs was significantly lower compared with that of the ds*GFP*‐injected control nymphs. The SRBSDV infection efficiency in the ds*GFP*‐, ds*VAMP7*‐, and ds*Vti1a*‐injected groups was 69.3%, 24.7%, and 23.3%, respectively (Figure S4). We also quantified the mRNA levels of *VAMP7*, *Vti1a*, and SRBSDV *P10* after injection; the mRNA levels of *VAMP7* and *Vti1a* were significantly lower in the haemolymph and salivary glands in the ds*VAMP7*‐ and ds*Vti1a*‐injected groups compared with the ds*GFP*‐injected group (Figure 6g,i), and the same was observed for the RNA levels of SRBSDV *P10* (Figure 6h,j). The above results showed that the virus titre was significantly lower in the haemolymph and salivary glands, suggesting that the downregulation of *VAMP7* or *Vti1a* restricts SRBSDV spread from the midgut to the haemolymph and to salivary glands.

To determine whether decreased expression of VAMP7 or Vti1a directly caused the decrease in virus accumulation in haemolymph, we injected the haemolymph from SRBSDV‐infected insects directly into virus‐free insects so that the virions could bypass the gut. At the same time, ds*VAMP7*, ds*Vti1a*, or ds*GFP* was also injected into insects. The haemolymph was then isolated from each group at 0 days after injection to detect the SRBSDV *P10* RNA levels. We found no difference in virus titre between the three groups (Figure S5a). At 4 days after injection, some virions were present in the haemocytes from the ds*VAMP7*‐, ds*Vti1a*‐, and ds*GFP*‐injected insects, as observed by LSCM (Figure S5b–d). After 4 more days, the haemolymph was isolated and analysed by quantitative reverse transcription PCR (RT‐qPCR). Although the mRNA levels of *VAMP7* and *Vti1a* were significantly lower in the haemolymph in the ds*VAMP7* and ds*Vti1a* groups compared with the ds*GFP* group (Figure S5e,g), the RNA level of SRBSDV *P10* in the ds*VAMP7* and ds*Vti1a* groups was not significantly different from the control levels (Figure S5f,h). These results suggested that downregulation of *VAMP7* or *Vti1a* did not directly affect SRBSDV accumulation in the haemolymph, but this treatment blocked release of virions from the gut to the haemolymph.

### 2.7 Effects of vesicle transport on acquisition and transmission of SRBSDV by WBPH

**FIGURE 7:**
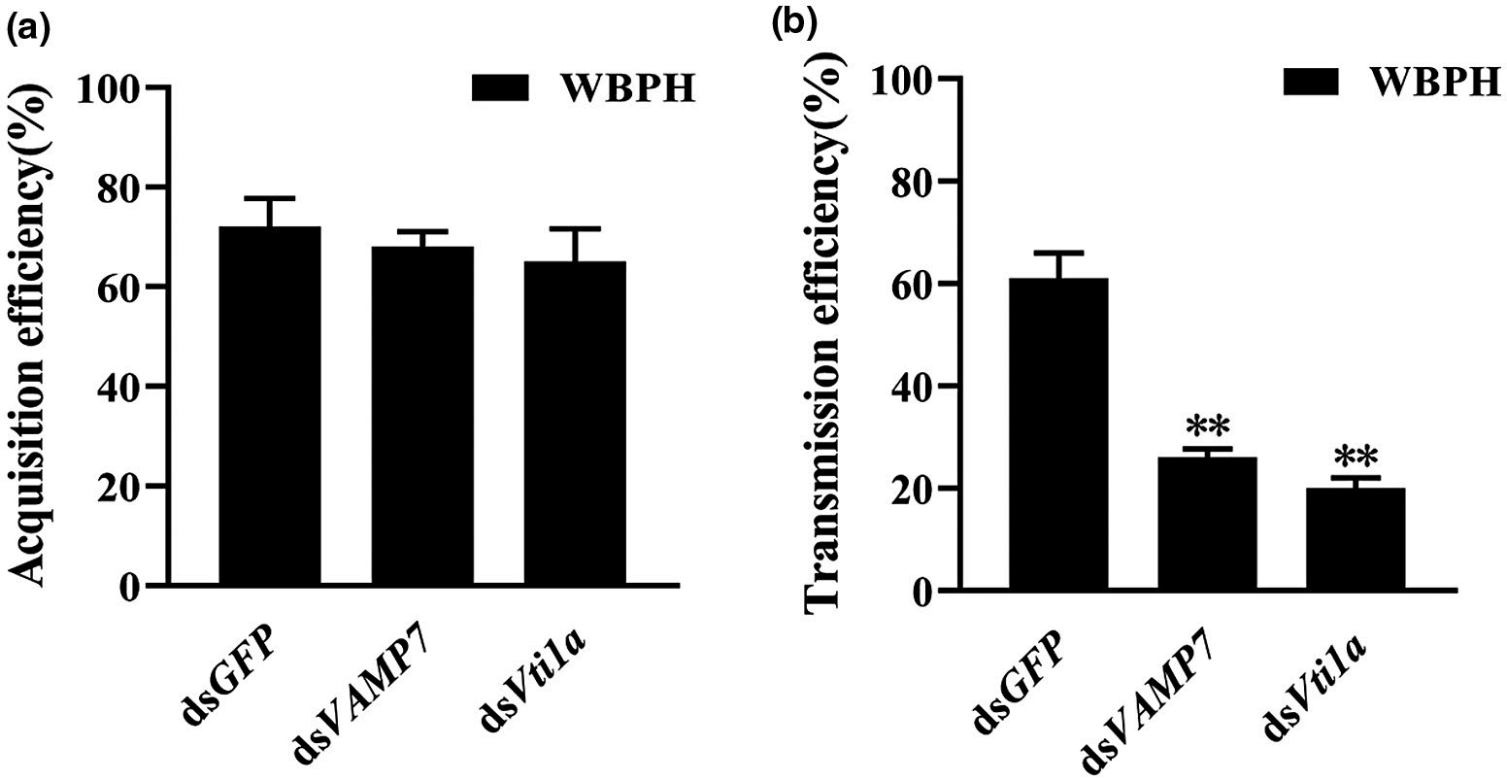
Acquisition and transmission efficiency after expression of VAMP7 or Vti1a was downregulated in white‐backed planthopper (WBPH) vector insects. (a) Acquisition efficiency of SRBSDV by insects after injection with ds*GFP*, ds*VAMP7*, or ds*Vti1a*. Total RNA was extracted from guts of WBPH to detect the virus using reverse transcription PCR. (b) Transmission efficiency of virus to rice seedlings by viruliferous WBPHs after injection with different dsRNAs. Data are presented as the mean ± *SEM* of three independent experiments (Student's *t* test, **p < 0.01)

When the expression of *VAMP7* or *Vti1a* was inhibited, the average SRBSDV acquisition efficiency did not differ significantly among the three groups (72% for the control, 68% for the ds*VAMP7*‐treated insects, and 65% for the ds*Vti1a*‐treated insects; mean ± *SEM* of three independent experiments, Student's *t* test, *p* > 0.05) (Figure 7a; Table S2). Thus, downregulation of *VAMP7* or *Vti1a* had no significant effect on SRBSDV entry and replication in the WBPH.

When we examined the influence of downregulation of *VAMP7* and *Vti1a* on virus transmission by WBPHs, the transmission efficiencies declined to 26% after ds*VAMP7* injection and to 20% after ds*Vti1a* injection compared with 61% for the control (mean ± *SEM* of three independent experiments, Student's *t* test, *p* < 0.01) (Figure 7b; Table S3). Downregulation of *VAMP7* or *Vti1a* inhibited virus transportation from the gut to salivary glands; thus, virus transmission from WBPHs to rice plants significantly decreased.

## 3 DISCUSSION

Vesicular trafficking is the main pathway for cellular uptake, endocytic transport, and secretion of macromolecules such as lipids, RNAs, membrane proteins, signalling molecules, and enzymes (Faure et al., 2006; Kajimoto et al., 2013; Novick et al., 1980; Vlahakis, 2006). Rotavirus, a reovirus, was found in extracellular vesicles, which were highly virulent units for virus transmission (Santiana et al., 2018). However, the involvement of intracellular vesicles of reoviruses has not been studied in great detail. In particular, there is little evidence about the mechanism of virion entry into vesicles. Here, we found numerous SRBSDV virions within vesicles that were close to the basal lamina in gut epithelial cells of the vector WBPH, suggesting that vesicles might be involved in the transport of viruses in and out of cells.

Interestingly, two SNARE proteins, VAMP7 and Vti1a, which interacted with each other, were confirmed to also interact with the outer major capsid protein P10 of SRBSDV. After infection and replication of the virus in epithelial cells, the colocalization pattern of the virus and the two proteins gradually moved from the cytoplasm to the cell membrane. These results indicated that VAMP7 and Vti1a are probably involved in the movement of the virus from inside to outside the cells. Furthermore, after knockdown of *VAMP7* or *Vti1a* at the mRNA level, the virus could not be detected in the muscle layer outside the epithelial cells. The viral titre was also significantly lower in the haemolymph and salivary glands when *VAMP7* or *Vti1a* expression was suppressed in insects that acquired virus from the plant. However, viral titre in the haemolymph of insects was unaffected when the insects were injected directly with the virus. Therefore, SRBSDV virions released from the gut cells need the assistance of VAMP7 and Vti1a. Because the SNARE complex that comprises VAMP7 and Vti1a has been shown to be involved in the transport of substances to the cell surface of HeLa and neuro2A cells (Flowerdew & Burgoyne, 2009), we suspect that the interaction of SRBSDV with VAMP7 and Vti1a via P10 might promote vesicle fusion to the cell membrane and subsequent release of the virus from the epithelial cells to the haemocoel.

The interaction between VAMP7 and Vti1a has been suggested to mediate intracellular vesicle fusion (Flowerdew & Burgoyne, 2009). Thus, when expression of *VAMP7* or *Vti1a* was inhibited in virus‐infected WBPH, vesicles were found less abundant than in the ds*GFP*‐treated insects, suggesting that without either VAMP7 or Vti1a, the vesicles might not fuse in the WBPH gut cells. In addition, we also found more fusing/fused vesicles in virus‐infected cells compared with the uninfected cells, probably because viral P10, by interacting with VAMP7 and Vti1a, may shorten the distance between these two proteins to facilitate their interaction and thus promote vesicle fusion. The fusion of these vesicles may facilitate the movement of the virus within the cells. Inhibition of either VAMP7 or Vti1a also led to the presence of virions in the cytoplasm but not in the vesicles of insect cells. Thus, both VAMP7 and Vti1a play a key role in virus entry into vesicles.

However, the viral titre did not change in the gut cells of vector insects after expression of *VAMP7* and *Vti1a* was inhibited, indicating that VAMP7 and Vti1a are not involved in viral entry and replication in the cells, perhaps because SRBSDV initially enters cells by clathrin‐dependent endocytosis (Danthi et al., 2010; Ehrlich et al., 2004). This process is not involved in the vesicle fusion mediated by VAMP7 and Vti1a. SRBSDV is also known to replicate in viroplasms in the cytoplasm without the need for vesicles (Jia, Chen, Zheng, et al., 2012); thus, vesicles and their related proteins VAMP7 and Vti1a also do not influence viral replication in the cells.

Although some studies have focused on the movement of reoviruses within and from cells, the underlying mechanisms have not been clarified (Chen et al., 2012; van Dongen et al., 2016; Mercer et al., 2010). The nonstructural protein P7‐1 of SRBSDV can form tubules to spread virions directly from the infected gut epithelial cells through the basal lamina to the haemocoel of WBPH (Liu et al., 2011). However, here we found a novel pathway for the intracellular and extracellular movement of SRBSDV and elucidated the mechanisms in more detail. Our study provided evidence that SRBSDV can use vesicles to move within the epithelial cells and be released from the cells. Virions of other reoviruses, such as rotaviruses, are cloaked by extracellular vesicles, which is the optimal unit for faecal–oral transmission (Santiana et al., 2018). However, the mechanism that enables these viruses to enter vesicles is not well understood, and even less is known about the role of vesicles in the transport of viruses. Recently, mammalian orthoreoviruses were found to be secreted from cells through the modification of lysosomal organelles, but the mechanisms enabling the virions to recognize and interact with lysosomes need to be clarified (de Castro et al., 2020). Importantly, we found that SRBSDV P10 can bind the SNARE proteins VAMP7 and Vti1a to enable entry of the viral particles into the vesicles, their intracellular spread via the vesicles, and dissemination from the gut epithelium into the haemolymph.

**FIGURE 8:**
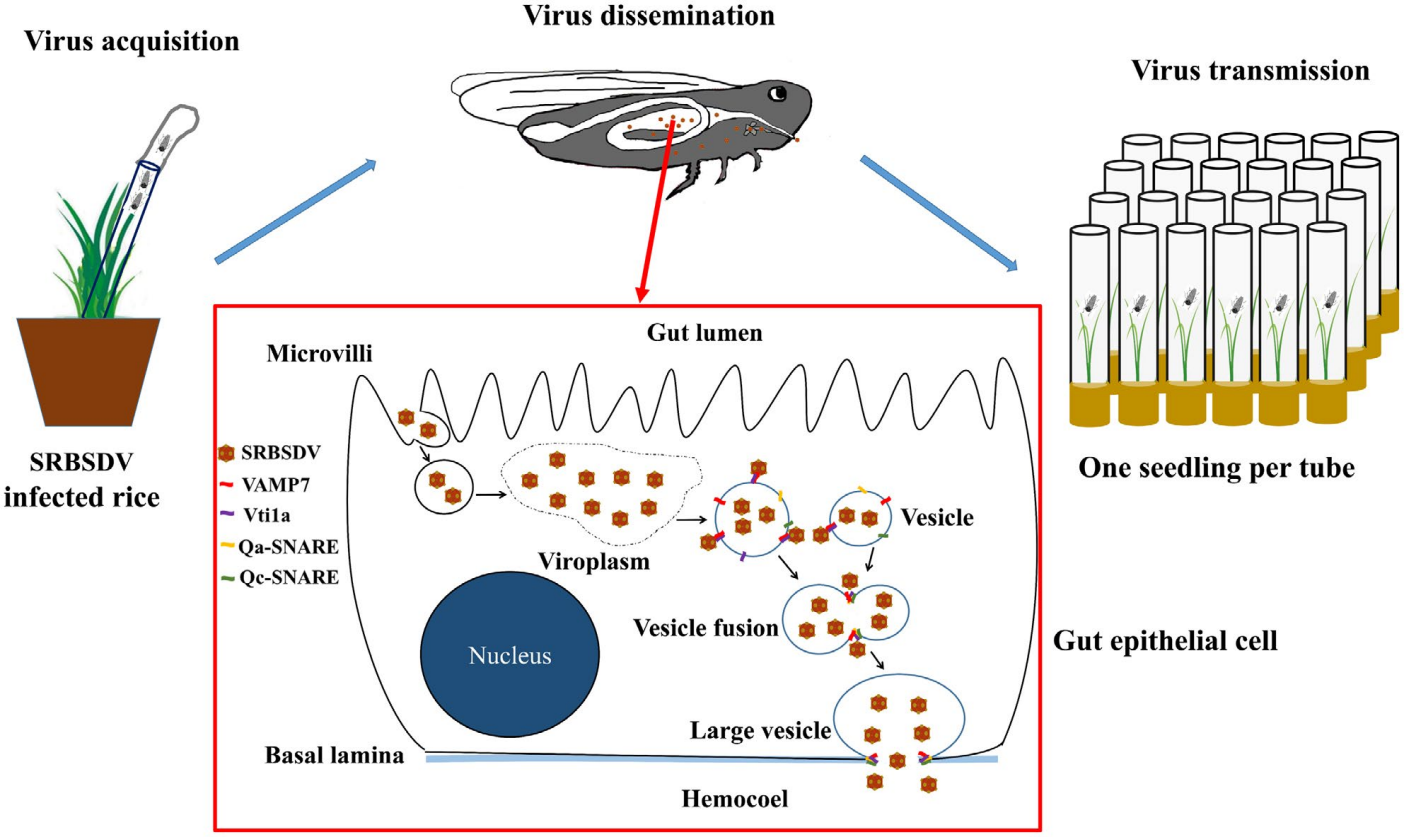
Dissemination pathway of SRBSDV in midgut epithelial cells. SRBSDV acquired by white‐backed planthoppers (WBPHs) commonly infects the insect cells by endocytosis and replicates in the viroplasm. The newly assembled virions enter the vesicles by binding VAMP7 and Vti1a of WBPHs through the interaction with SRBSDV P10. Then, these vesicles fuse to form a large vesicle, which is also promoted by the interaction of VAMP7, Vti1a, and P10. The vesicles fuse with the cell membrane to release large quantities of virions from the infected cells into the haemolymph, evidently overcoming the insect's midgut barrier. After infecting the salivary gland, virions are transmitted in saliva during feeding on plants

Our data provide a hypothetical framework to explain the hijacking of the SNARE complex by a reovirus to enable passage through the midgut, a key step for virus transmission. After acquisition by WBPH, virions enter midgut epithelial cells by endocytosis and then replicate in the viroplasm in the cytoplasm. The virions are then packaged in vesicles through the interaction of the major outer viral capsid protein P10 with SNARE proteins VAMP7 and Vti1a, which execute membrane fusion. Viral P10 might promote fusion of virion‐containing vesicles into a large vesicle for transport by bringing VAMP7 and Vti1a closer together. This vesicle trafficking facilitates movement of numerous virions so that SRBSDV can be rapidly released from the epithelial cells and thus cross the insect's midgut barrier into the haemolymph (Figure 8). This strategy of a reovirus using SNARE proteins for intracellular trafficking in insect vector cells is novel.

Most arthropod‐borne viruses are transmitted by specific vector insects (Bragard et al., 2013; Hogenhout et al., 2008; Mauck et al., 2012). Interestingly, some insects such as *Aedes aegypti* cannot acquire eastern equine encephalomyelitis virus (genus *Alphavirus*, family *Togaviridae*) by sucking viruliferous blood, but they can transmit this virus after a viral suspension is directly injected into the haemocoel of the insect (Merrill & Tenbroeck, 1935). Many plant viruses can also be transmitted by insects that are not natural hosts when the virus is injected directly into the insect's abdomen (Nault et al., 1980; Yao et al., 2019). Our findings show that the virus can utilize vesicles by binding to the SNARE proteins VAMP7 and Vti1a to cross the barrier of midgut epithelial cells and suggest that other animal or plant viruses might also be transported by vesicles inside cells. The SNARE proteins VAMP7 and Vti1a can be regarded as targets for developing novel strategies for virus disease control.

## 4 EXPERIMENTAL PROCEDURES

### 4.1 WBPH rearing and SRBSDV maintenance

WBPHs, originally captured in Nanjing, China, were reared in glass beakers with rice seedlings in incubators at 28 °C with 16 hr light and 8 hr dark in our laboratory. We moved the insects onto fresh rice seedlings every week. SRBSDV‐infected rice plants were originally provided by Prof. G. Zhou (South China Agricultural University), and newly infected plants were grown in a greenhouse. Infected rice plants were routinely tested every 2 months for virus, especially before allowing nonviruliferous WBPH to feed.

### 4.2 Yeast two‐hybrid assay

The split‐ubiquitin Y2H system (Dualsystems Biotech) was used to assess interactions among SRBSDV P10, VAMP7, and Vti1a (Liu et al., 2015). Yeast strain NMY51 was shake‐cultured overnight at 30 °C in 50 ml yeast peptone dextrose adenine (YPDA) broth until the optical density at 546 nm (OD_546_) reached 0.6–0.8. After centrifugation, the NMY51 cells were pelleted and resuspended in 2.5 ml water. Then the yeast cells were cotransformed with different pairings of SRBSDV P10, VAMP7, and Vti1a. Large T + P53 was used as the positive control; large T + pPR3‐N served as the negative control. The transformed yeast cells were then plated onto 10‐cm diameter plates of selection media (double dropout, DDO: SD−Leu−Trp; quadruple dropout, QDO: SD−Ade−His−Leu−Trp) with 20 mM 3‐aminotriazole. After 4 days at 30 °C, the strength of the protein–protein interaction between the bait and prey was evaluated using the HTX High‐throughput β‐Galactosidase Assay Kit (Dualsystems Biotech).

### 4.3 Cell transfection

Recombinant SRBSDV *P10*, *VAMP7*, and *Vti1a* were amplified by PCR with specifically designed primers (Table S4) and cloned into the Bac‐to‐Bac baculovirus expression plasmids. Then the cells were transfected using Cellfectin II (Invitrogen) according to the instructions (Qin et al., 2018). About 2 × 10^6^ cells were added to each well of a six‐well culture dish, and after 30 min, a mixture of 2 μg recombinant Bac plasmids and 8 μl Cellfectin II was added to the wells, which were incubated at 27 °C for 5 hr. Then the transfection mixture was replaced with growth medium. After 2 days, the transfected *Spodoptera frugiperda* (Sf9) cells were observed by LSCM.

### 4.4 Coimmunoprecipitation

The plate with Sf9 cells and only recombinant baculovirus of the prey served as the negative control, and the plate with Sf9 cells, the bait, and the prey was the experimental group. After 2 hr, the medium in the two plates was removed, the cells were washed with phosphate‐buffered saline (PBS), fresh growth medium was added, and plates were incubated at 27 °C. All cells were then collected at 48 hr and ground in liquid nitrogen. After 1 ml of protein extraction buffer (50 mM Tris‐HCl, pH 7.5, 150 mM NaCl, 4 mM MgCl_2_, 5 nM dithiothreitol [DTT], and 1% NP‐40 [vol/vol]) was added, samples were incubated for 20 min at 4 °C and centrifuged at 9,500 × g for 25 min, and the supernatant was collected. For eliminating protein that could bind nonspecifically to Pierce protein A/G agarose beads (Thermo Scientific), 50 μl of beads was added to the supernatant, and the mixture was incubated at 4 °C for 1 hr with shaking and centrifuged at 100 × g  for 5 min. The supernatant was collected, 1 μl of anti‐bait antibody was added, and the solution was incubated with shaking at 4 °C for 2 hr. Then 100 μl of beads was added. After incubation at 4 °C with shaking for 2 hr, the mixture was centrifuged at 100 × g at 4 °C for 5 min, the supernatant was discarded, and 1 ml elution buffer (50 mM Tris‐HCl, pH 7.5, 150 mM NaCl, 4 mM, MgCl_2_, 2 nM DTT, and 1% NP‐40 [vol/vol]) was added to rinse the beads for 10 min. The mixture was centrifuged at 100 × g at 4 °C for 5 min and washed three times by centrifuging at 100 × g, and the supernatant was discarded. The bead mixture was combined with 200 μl of 2× sodium dodecyl sulphate (SDS)‐polyacrylamide gel electrophoresis (PAGE) loading buffer and incubated in boiling water for 10 min. Proteins were separated by SDS‐PAGE and then transferred from the gel to a nitrocellulose membrane, which was incubated with anti‐prey antibody to detect proteins.

### 4.5 Antibodies and reagents

The anti‐SRBSDV antibody was graciously provided by Prof. J. Wu (Zhejiang University). The anti‐VAMP7 antibody and the anti‐Vti1a antibody were produced by GenScript. Horseradish peroxidase (HRP)‐conjugated secondary goat anti‐rabbit and goat anti‐mouse antibodies were purchased from KPL. Alexa Fluor 633 phalloidin was obtained from Invitrogen, and 4',6‐diamidino‐2‐phenylindole (DAPI) was purchased from Abcam.

### 4.6 Immunofluorescence microscopy

Sf9 cells previously fixed on cover slips or freshly excised WBPH tissues were incubated in 4% (vol/vol) paraformaldehyde in PBS for 2 hr at room temperature and washed three times with PBS. The samples were subsequently permeabilized in 2% (vol/vol) Triton X‐100 for 30 min at room temperature and incubated overnight at 4 °C with Dylight 488‐conjugated anti‐SRBSDV (green) and Dylight 549‐conjugated anti‐VAMP7 or Dylight 549‐conjugated anti‐Vti1a (red) and then with DAPI (for Sf9 cells) or Alexa Fluor 633 phalloidin (for WBPH tissues) for 2 hr at room temperature. All samples were visualized with a LSM880 laser scanning confocal microscope (Zeiss), and the images were saved using ZEN 2011 blue light. The data were analysed by ImageJ v. 1.52 (National Institutes of Health).

### 4.7 Immunoelectron microscopy

WBPH nymphs were put onto SRBSDV‐infected rice plants to acquire the virus and then moved onto healthy rice seedlings after a 2‐day AAP. The midguts were then excised on different days, fixed in 2% (vol/vol) paraformaldehyde and 2% (wt/vol) osmium tetroxide in PBS for 2 hr, dehydrated in an ethanolic series (30%, 50%, 70%, 90%, 95%, and 100%), and finally embedded in LR Gold Resin (Sigma). The specimens were sectioned with an ultramicrotome (Leica) and then blocked for 30 min in PBS blocking buffer. The sections were stained using 2% uranyl acetate (dissolved in 50% ethanol) and alkaline lead citrate (0.08 M Pb(NO_3_)_2_, 0.12 M C_6_H_5_Na_3_O_7_.2H_2_O) for 5–10 min or incubated at room temperature with the primary antibody followed by incubation with a gold‐conjugated secondary antibody (the gold particles for VAMP7 are 10 nm in diameter and the gold particles for Vti1a are 5 nm in diameter), and then stained. The sections were observed with a transmission electron microscope at an acceleration voltage of 80 kV. The isolated haemolymph was negatively stained with 1%–2% (wt/vol) phosphotungstic acid, pH 6.5–7.0, for 15–30 s for electron microscopy.

### 4.8 Western blotting

Total protein was extracted from individual insect tissues using the Insect Total Protein Extraction Kit (Beibo). After the mixture was centrifuged at 12,000 ×*g* for 30 min at 4 °C, the supernatant was used for western blotting with anti‐VAMP7, anti‐Vti1a, anti‐SRBSDV, anti‐c‐myc tag, and anti‐His tag antibodies.

### 4.9 RT‐qPCR

cDNA was synthesized from 1 μg total RNA from WBPH using a FastQuant RT kit with gDNase (TIANGEN). Total RNA was incubated at 42 °C for 3 min with a gDNA‐removal mixture and incubated first at 42 °C for 15 min with reverse transcription buffer and then at 95 °C for 3 min to terminate the reaction. The qPCR was carried out using a SuperReal PreMix plus (SYBR Green) kit (TIANGEN) and an ABI 7500 thermocycler (Applied Biosystems) with the following PCR programme: 94 °C for 15 min, followed by 40 cycles of 95 °C for 10 s and 60 °C for 32 s. Fluorescence was measured at the end of every 60 °C extension phase. The *β‐actin* gene expression was used for normalization. Relative expression was calculated using the 2^−ΔΔ*C*t^ method. The experiments were done three times independently.

### 4.10 RNA interference assay

The T7 RiboMAX express RNAi System (Promega) was used to synthesize ds*GFP*, ds*VAMP7*, and ds*Vti1a*. More than 200 nonviruliferous third‐instar WBPH nymphs were injected with 23 nl of ds*GFP* (3 μg/μl), ds*VAMP7* (3 μg/μl), or ds*Vti1a* (3 μg/μl) using an Auto‐Nanolitre injector (Drummond). Then the injected insects were reared on healthy rice plants for 2 days, followed by a 2‐day AAP on SRBSDV‐positive rice plants. The midgut was then excised from 30 insects to check each one for the influence of the respective dsRNAs on SRBSDV entry, and the other insects were moved onto healthy rice seedlings to check the acquisition rate after 7 days. Then 200 newborn third‐instar WBPH nymphs that were produced on SRBSDV‐positive rice plants were injected with 23 nl ds*GFP* (3 μg/μl), ds*VAMP7* (3 μg/μl), or ds*Vti1a* and reared on fresh nonviruliferous rice seedlings. From 50 nymphs in each treatment group, protein was extracted and expression levels of VAMP7 or Vti1a and SRBSDV P10 were analysed by western blot to check the influence of the treatments on SRBSDV replication.

To explore the influence of the treatments on dissemination in different tissues, we injected 300 third‐instar WBPH nymphs that had finished a 2‐day AAP with 23 nl ds*GFP* (3 μg/μl), ds*VAMP7* (3 μg/μl), or ds*Vti1a* (3 μg/μl) and reared them on healthy rice seedlings. Then the midgut and salivary glands were excised from 30 nymphs from each treatment group to observe each one for fluorescence of SRBSDV by LSCM. Similarly, guts, haemolymph, and salivary glands were excised from 50 nymphs in each group and RNA was extracted to quantify *VAMP7*, *Vti1a*, and SRBSDV *P10* mRNA levels using RT‐qPCR. At the same time, the guts were excised from 100 nymphs, and VAMP7, Vti1a, and SRBSDV P10 protein levels were quantified by western blot. To test transmission efficiency, we moved 100 other nymphs to healthy rice seedlings, and 7 days later placed each nymph on its own rice seedling for 2 days. After 15 days, we tested individual transmission efficiency of SRBSDV to the plant using RT‐PCR. For virus injection, the haemolymph in insects with SRBSDV was isolated from the wounds in forelegs that had been severed at the coxatrochanter joint. The haemolymph was then injected into WBPHs with ds*GFP* (3 μg/μl), ds*VAMP7* (3 μg/μl), or ds*Vti1a* (3 μg/μl). After 4 days, the haemolymph was removed from the injected insects for immunofluorescence microscopy and RT‐qPCR detection.

## CONFLICT OF INTEREST

The authors declare that they have no conflicts of interest.

## Supporting information

**FIGURE S1** Structural analysis of VAMP7 and Vti1a. (a) Transmembrane domain and signal peptide of VAMP7. (b) Transmembrane domain and signal peptide of Vti1a. The structures of VAMP7 and Vti1a were predicted by TMHMM and SignalP v. 4.1 software

**FIGURE S2** SRBSDV P7‐1 did not interact with VAMP7 or Vti1a. (a) Yeast strain NMY51 was co‐transformed with PDHB1‐SRBSDV P7‐1 and either pPR3N‐VAMP7 or pPR3N‐Vti1a. Yeast cells, diluted from 10^−1^ to 10^−4^, were plated onto DDO (SD−Trp−Leu) and QDO (SD−Trp−Leu−His−Ade) medium. (b) Clones grown on DDO were selected for the β‐galactosidase assay. Large T + P53 was used as the positive control; PDHB1‐SRBSDV P7‐1 + pPR3N served as the negative control

**FIGURE S3** Number of fluorescent puncta of SRBSDV P10, VAMP, and Vti1a in midgut epithelial cells. (a) Number of fluorescent puncta of SRBSDV P10 and VAMP7. (b) Number of fluorescent puncta of SRBSDV P10 and VAMP7 that colocalized in cells. (c) Number of fluorescent puncta of SRBSDV P10 and VAMP7 that colocalized on the cell membrane. (d) Number of fluorescent puncta of SRBSDV P10 and Vti1a. (e) Number of fluorescent puncta of SRBSDV P10 and Vti1a that colocalized in cells. (f) Number of fluorescent puncta of SRBSDV P10 and Vti1a that colocalized on the cell membrane. Student’s *t*‐test, NS: not significant (*p* > .05), **p < .01

**FIGURE S4** SRBSDV infection efficiency into salivary glands of viruliferous white‐backed planthoppers after down‐regulation of VAMP7 or Vti1a. Data for SRBSDV infection efficiency of salivary glands in dsVAMP7‐ or dsVti1a‐injected nymphs were analysed at 8 days after virus acquisition using Student’s *t*‐test (*p < .05). Means ± *SEM* are shown for three independent experiments

**FIGURE S5** VAMP7 or Vti1a did not affect viral titre in the haemolymph. (a) RNA levels of SRBSDV *P10* in haemolymph from nymphs injected with ds*GFP*, ds*VAMP7*, or ds*Vti1a* at 0 days after virion injection. (b–d) The haemolymph with SRBSDV was microinjected into virus‐free white‐backed planthoppers. Haemocytes from nymphs injected with (a) ds*GFP*, (b) ds*VAMP7*, or (c) ds*Vti1a* were incubated with anti‐SRBSDV antibody labelled with Dylight 488 (green). Scale bars, 25 µm. (e–h) The mRNA levels of *VAMP7* and *Vti1a* and RNA levels of SRBSDV *P10* in haemolymph from nymphs injected with ds*GFP*, ds*VAMP7*, or ds*Vti1a* as quantified using quantitative reverse transcription PCR 4 days after virion injection. Mean ± *SEM* of three independent experiments, Student’s *t*‐test (**p < .01)

**TABLE S1** Sequences of *VAMP7* and *Vti1a* in the white‐backed planthopper

**TABLE S2** SRBSDV acquisition efficiency by white‐backed planthoppers injected with ds*GFP,* ds*VAMP7*, or ds*Vti1a*

**TABLE S3** SRBSDV transmission efficiency by white‐backed planthoppers injected with ds*GFP,* ds*VAMP7*, or ds*Vti1a*

**TABLE S4** Primers used in this study

## Data Availability

Data on the vesicle membrane proteins VAMP7 and Vti1a are available at NCBI at https://www.ncbi.nlm.nih.gov/genbank/ under accession numbers MN764900 (VAMP7) and MN764901 (Vti1a).
