# Supplementary material for: Southern rice black‐streaked dwarf virus hijacks SNARE complex of its insect vector for its effective transmission to rice
Source: Mol Plant Pathol. 2021 Aug 13;22(10):1256–70. doi: 10.1111/mpp.13109 (PMC8435234; doi:10.1111/mpp.13109)
Supplement: Supplementary file 1 — FIGURE S1 Structural analysis of VAMP7 and Vti1a. (a) Transmembrane domain and signal peptide of VAMP7. (b) Transmembrane domain and signal peptide of Vti1a. The structures of VAMP7 and Vti1a were predicted by TMHMM and SignalP v. 4.1 software [file MPP-22-1256-s004.docx]

**
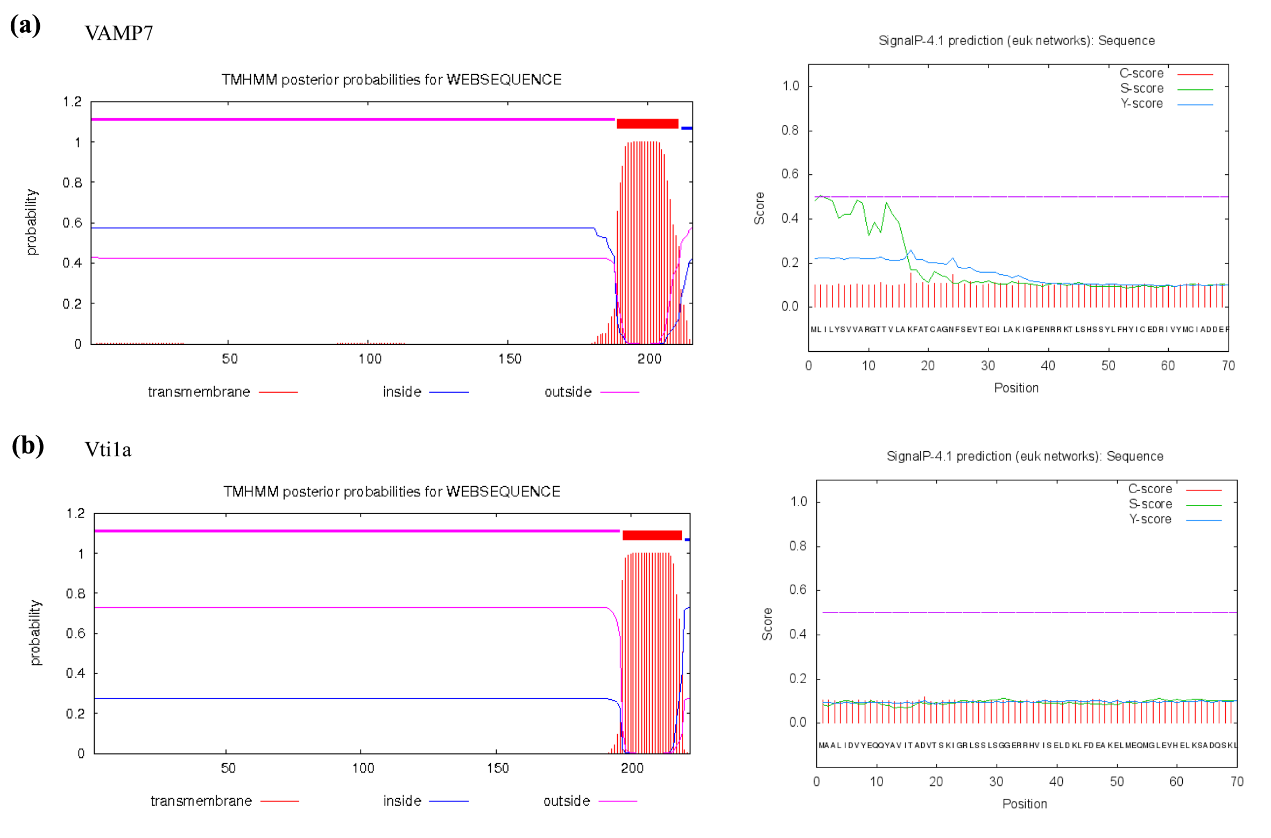
**

**Figure S1** Structural analysis of VAMP7 and Vti1a. (a) Transmembrane domain and signal peptide of VAMP7. (b) Transmembrane domain and signal peptide of Vti1a. The structure of VAMP7 and Vti1a were predicted by TMHMM and SignalP 4.1 softwares.
