# Supplementary material for: Southern rice black‐streaked dwarf virus hijacks SNARE complex of its insect vector for its effective transmission to rice
Source: Mol Plant Pathol. 2021 Aug 13;22(10):1256–70. doi: 10.1111/mpp.13109 (PMC8435234; doi:10.1111/mpp.13109)
Supplement: Supplementary file 2 — FIGURE S2 SRBSDV P7‐1 did not interact with VAMP7 or Vti1a. (a) Yeast strain NMY51 was co‐transformed with PDHB1‐SRBSDV P7‐1 and either pPR3N‐VAMP7 or pPR3N‐Vti1a. Yeast cells, diluted from 10−1 to 10−4, were plated onto DDO (SD−Trp−Leu) and QDO (SD−Trp−Leu−His−Ade) medium. (b) Clones grown on DDO were selected for the β‐galactosidase assay. Large T + P53 was used as the positive control; PDHB1‐SRBSDV P7‐1 + pPR3N served as the negative control [file MPP-22-1256-s007.docx]

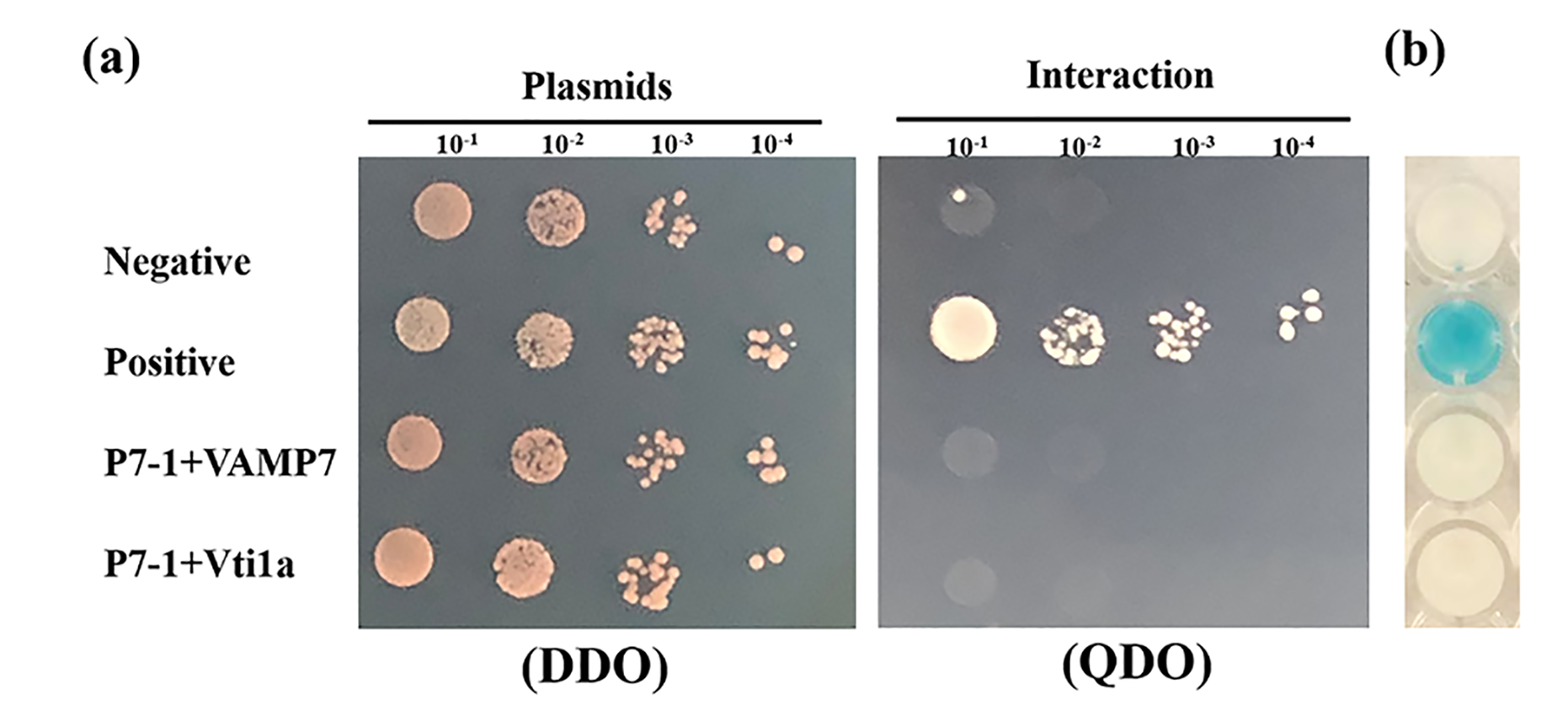


**Figure S2** SRBSDV P7-1 did not interact with VAMP7 or Vti1a. (a) Yeast strain NMY51 was cotransformed with PDHB1-SRBSDV P7-1 and either with pPR3N-VAMP7 or pPR3N-Vti1a. Yeast cells, diluted from 10^-1^ to 10^-4^, were plated onto DDO (SD-trp-leu) and QDO (SD-trp-leu-his-ade) medium. (b) Clones grown on DDO were selected for β-galactosidase activity assay. Large T + P53 was used as the positive control; PDHB1-SRBSDV P7-1 + pPR3N served as the negative control.
