# Supplementary material for: Southern rice black‐streaked dwarf virus hijacks SNARE complex of its insect vector for its effective transmission to rice
Source: Mol Plant Pathol. 2021 Aug 13;22(10):1256–70. doi: 10.1111/mpp.13109 (PMC8435234; doi:10.1111/mpp.13109)
Supplement: Supplementary file 3 — FIGURE S3 Number of fluorescent puncta of SRBSDV P10, VAMP, and Vti1a in midgut epithelial cells. (a) Number of fluorescent puncta of SRBSDV P10 and VAMP7. (b) Number of fluorescent puncta of SRBSDV P10 and VAMP7 that colocalized in cells. (c) Number of fluorescent puncta of SRBSDV P10 and VAMP7 that colocalized on the cell membrane. (d) Number of fluorescent puncta of SRBSDV P10 and Vti1a. (e) Number of fluorescent puncta of SRBSDV P10 and Vti1a that colocalized in cells. (f) Number of fluorescent puncta of SRBSDV P10 and Vti1a that colocalized on the cell membrane. Student’s t‐test, NS: not significant (p > .05), **p < .01 [file MPP-22-1256-s009.docx]

**
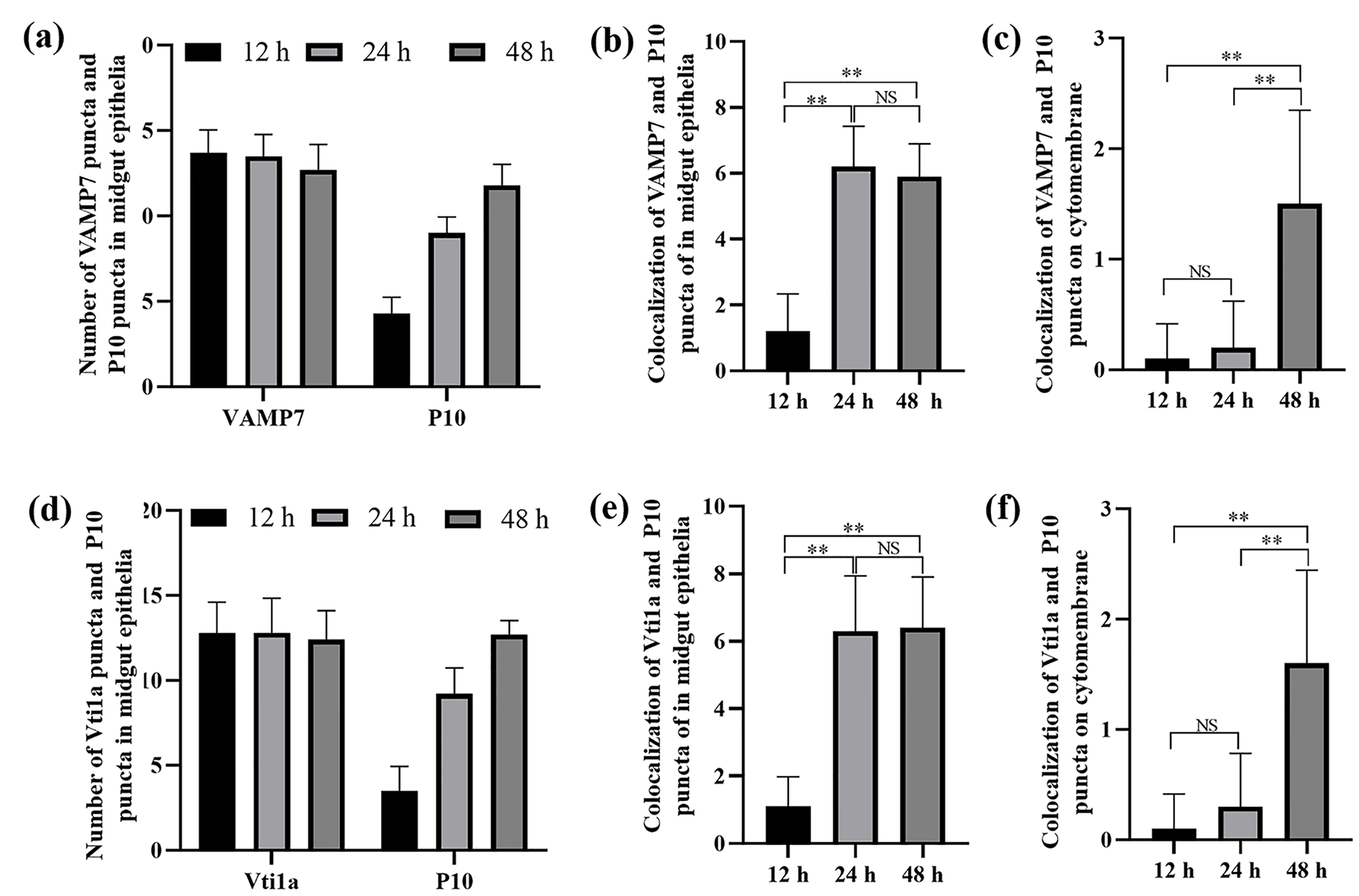
**

**Figure S3** Number of fluorescent puncta of SRBSDV P10, VAMP7 and Vti1a in midgut epithelial cells. (a) Number of fluorescent puncta of SRBSDV P10 and VAMP7. (b) Number of fluorescent puncta of SRBSDV P10 and VAMP7 that colocalized in cells. (c) Number of fluorescent puncta of SRBSDV P10 and VAMP7 that colocalized on cytomembrane of cells. (d) Number of fluorescent puncta of SRBSDV P10 and Vti1a. (e) Number of fluorescent puncta of SRBSDV P10 and Vti1a that colocalized in the cells. (f) Number of fluorescent puncta of SRBSDV P10 and Vti1a that colocalized on cytomembrane of cells. (NS: not significant, ***P* < 0.01, Student’s t-test)
