# Supplementary material for: Southern rice black‐streaked dwarf virus hijacks SNARE complex of its insect vector for its effective transmission to rice
Source: Mol Plant Pathol. 2021 Aug 13;22(10):1256–70. doi: 10.1111/mpp.13109 (PMC8435234; doi:10.1111/mpp.13109)
Supplement: Supplementary file 4 — FIGURE S4 SRBSDV infection efficiency into salivary glands of viruliferous white‐backed planthoppers after down‐regulation of VAMP7 or Vti1a. Data for SRBSDV infection efficiency of salivary glands in dsVAMP7‐ or dsVti1a‐injected nymphs were analysed at 8 days after virus acquisition using Student’s t‐test (*p < .05). Means ± SEM are shown for three independent experiments [file MPP-22-1256-s006.docx]

**
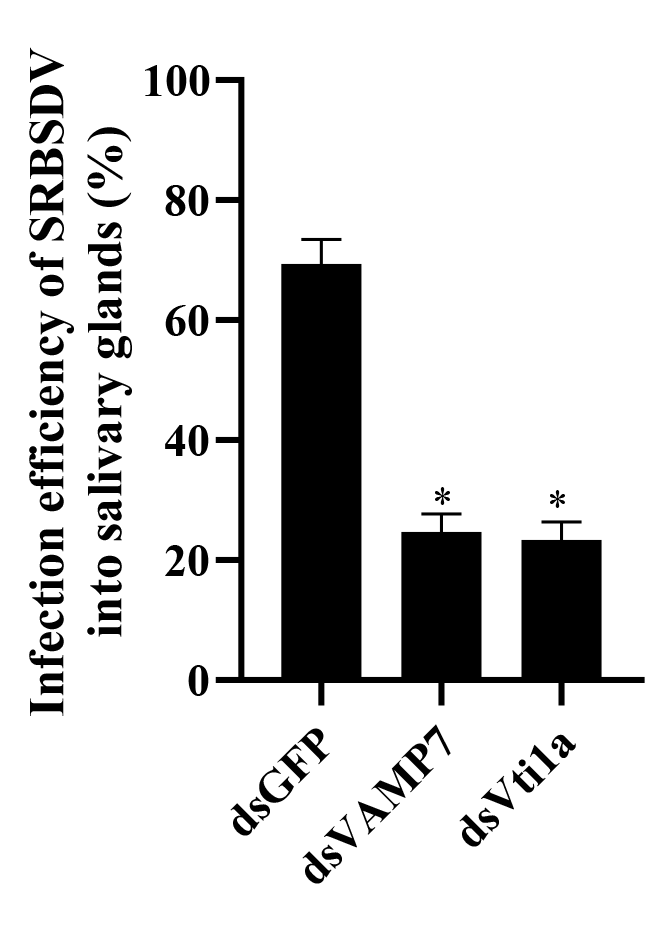
**

**Figure S4** SRBSDV infection efficiency into salivary glands of viruliferous WBPHs after expression of VAMP7 or Vti1a inhibited. Data for SRBSDV infection efficiency of salivary glands in dsVAMP7- or dsVti1a-injected nymphs was analyzed at 8d after virus acquisition using Student’s *t*-test (**P* < 0.05). Means ± SEM are shown for three independent experiments.
