# Supplementary material for: Southern rice black‐streaked dwarf virus hijacks SNARE complex of its insect vector for its effective transmission to rice
Source: Mol Plant Pathol. 2021 Aug 13;22(10):1256–70. doi: 10.1111/mpp.13109 (PMC8435234; doi:10.1111/mpp.13109)
Supplement: Supplementary file 5 — FIGURE S5 VAMP7 or Vti1a did not affect viral titre in the haemolymph. (a) RNA levels of SRBSDV P10 in haemolymph from nymphs injected with dsGFP, dsVAMP7, or dsVti1a at 0 days after virion injection. (b–d) The haemolymph with SRBSDV was microinjected into virus‐free white‐backed planthoppers. Haemocytes from nymphs injected with (a) dsGFP, (b) dsVAMP7, or (c) dsVti1a were incubated with anti‐SRBSDV antibody labelled with Dylight 488 (green). Scale bars, 25 µm. (e–h) The mRNA levels of VAMP7 and Vti1a and RNA levels of SRBSDV P10 in haemolymph from nymphs injected with dsGFP, dsVAMP7, or dsVti1a as quantified using quantitative reverse transcription PCR 4 days after virion injection. Mean ± SEM of three independent experiments, Student’s t‐test (**p < .01) [file MPP-22-1256-s003.docx]

**
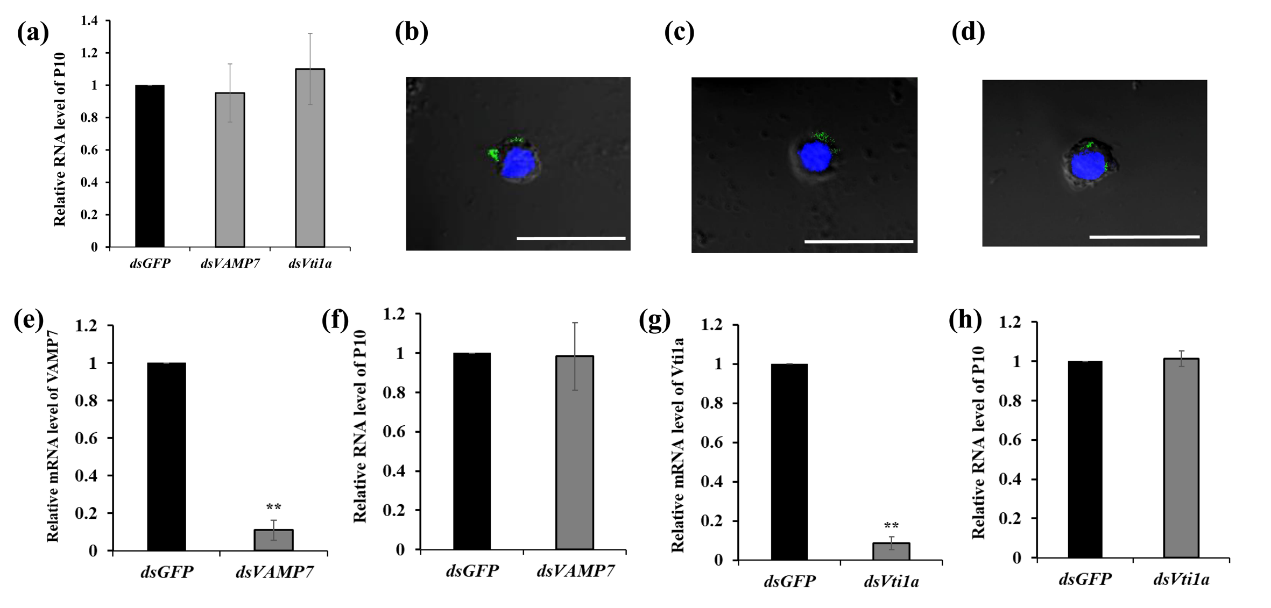
**

**Figure S5** VAMP7 or Vti1a did not affect viral titer in the hemolymph. (a) RNA level of SRBSDV P10 in hemolymph from nymphs injected with ds*GFP*, ds*VAMP7* or ds*Vti1a* were detected at 0 d after virions injected. (b-d) The hemolymph with SRBSDV was microinjected into virus-free white-backed planthoppers. Hemocytes from nymphs injected with ds*GFP* (a), ds*VAMP7* (b) or ds*Vti1a* (c) were incubated with anti-SRBSDV antibody labeled with Dylight 488 (green). Scale bars, 25 µm. (e-h) The mRNA level of VAMP7 and Vti1a and RNA level of SRBSDV P10 in hemolymph from nymphs injected with ds*GFP*, ds*VAMP7* or ds*Vti1a* quantified using RT-qPCR by 4d after virions injected. Mean ± SEM of three independent experiments, Student’s *t*-test (***p* < 0.01).
