# Supplementary material for: Southern rice black‐streaked dwarf virus hijacks SNARE complex of its insect vector for its effective transmission to rice
Source: Mol Plant Pathol. 2021 Aug 13;22(10):1256–70. doi: 10.1111/mpp.13109 (PMC8435234; doi:10.1111/mpp.13109)
Supplement: Supplementary file 6 — TABLE S1 Sequences of VAMP7 and Vti1a in the white‐backed planthopper [file MPP-22-1256-s002.docx]

**Table S1. Sequence of *VAMP7* and *Vti1a* in white-backed planthopper**

| Gene | Sequence |
| --- | --- |
| ***VAMP7*** | atctcactttcatttgattaaatcgaataaaa**atg**cttatcctatacagtgtagttgcaagagggaccacggttctggcaaaatttgcaacatgcgctggaaatttttccgaggtgacagaacaaatcctggcgaaaattggtccagaaaacaggaggaagactctctctcacagtagctatttattccattacatatgtgaggacagaattgtttacatgtgtattgcggatgatgagtttgaacgaccaagagcgtttttatacttgaatgaaataaaaaagaggttccgttcaacatatggcagcagagctgacacggccattgcttatgccatgaactcagagtttggccctattttaggaaatgaaatgaaatactattcagaatcgaaggatattgacacaatttcacgagtacatggcgaattggatgaattgaaagatataatggttcacaatattgataacattgctatgagagatgaacgcctcgaattgctggttgataaaacagaaaatctaacagccagctctgtcacattccgagcaactagtagaaatttacagcgagctctcttctggaaaaatatgaaactctatatgataatctcagctattgttttggtaataatttatttcatcttagctatgtcttgtggatcgactttgtccagttgc**tga**aagactacagagtaacaatcacctgtacggtttaattataatccgatgtattcttcagtaaagagtcttccttgtgatatgttcaatgataaatttatactattcttctaaagtagcatgtaaaacataagctacggtactgtattttttattttatccttatttcacggttcaattaatcaccagaaattatttgaataatccaatcatattcttaattattatatccataaacaaatggtaatgtgactgtatccactgtattaaataatagattgcaataaatttcaattaagagttttttactgcagtttgagtaatacaaaatagatccgttttatattagttgtttatatttattcacagattgttttattgtcagagaagtgaaacattgtatttgcataaggaatgtatgatagatacctatgaagatattattttgaattattctattttcttgataaatattatgttcataaacaaagggaaatgtaaatgtattgtaatccactgttttagatcatagattgtaataaatttctattgaagtacggttttttagattgcagtttcagtaatacaaaatagaaccgttttttattagttgcataaatttgaaaatatatatttctataaaaatattgttttattatcggagaattgaaacaatttatttgcataatgaaaataaaaaaaaaaaaaaaaaaaaaaaaaaaaa |
| ***Vti1a*** | aatgaaaaattattaataagttcaaactaatttagtagcctaaaacattagatt**atg**gctgctttaattgatgtttatgagcagcaatatgcagtcatcacagcagatgttacttcaaagattggaagattatcatctctttctggaggagaacgacgtcatgttatttcggaattggataagctgtttgatgaagccaaagagcttatggaacaaatgggccttgaagttcacgagttgaaatcagcagatcaatcaaaacttaagaacagaattgaaagttataaggcagaactgaaaagactagaacaagaatttactaatgctaaaaagaatatcaactcacaaaatggctacagcgataggatagaactgtatagcgaaagtataagcgtcaatgaggaacagaagcaaaggcttttggacaatgctgaaatggttgagcgcactggcaagaaattgactgctggctatcaagtgcttttggagactgaagatattggcaatcaagttctcagggacttgcattcgcagagggaaacgatacagaaatcgaggtctaggttaagagaaacaaacgccgagctgggaagaagtaacagaatcatcaattcgatgatcaacagatctctgcaacacaggttcatcttgacagctatcgccatcgtgttctgccttgtggtcgtgatcagcatctacatatcagctactcgctctcga**taa**actgcacttgtcaccatttagtactctactcggtgtatttcctaatcattctataattaaataatttattcactgctctagtatgaaattaaactaactatcattgaattgaactatcactgttgaaacaaacacctagtaaattattaattgtgtaaaagacaaaggacttcaatagctgtctgcctctctccgtcttaatataaaatgatctaataaaggattatctcatgctcaatatatgggtatcgtatgtgactatgattcttgatatcagtatttgaaaatgcattcatattttcctagaataataacttacgtctatcattgcacagtatattgtactaatttcataactcacatcatattgtttatcattttagatattttaaagtctgattgtgacttaattgtaacgaagatcattctggagctataaaatacttgagttattccaatatcttaattgctattgcaagaaaatgttatttgttttgtatgctgaatctattaaacgatagaaatctatgaaaagtttggat |
