# Supplementary material for: Southern rice black‐streaked dwarf virus hijacks SNARE complex of its insect vector for its effective transmission to rice
Source: Mol Plant Pathol. 2021 Aug 13;22(10):1256–70. doi: 10.1111/mpp.13109 (PMC8435234; doi:10.1111/mpp.13109)
Supplement: Supplementary file 7 — TABLE S2 SRBSDV acquisition efficiency by white‐backed planthoppers injected with dsGFP, dsVAMP7, or dsVti1a [file MPP-22-1256-s008.docx]

**Table S2. SRBSDV acquisition efficiency by WBPH injected with ds*GFP,* ds*VAMP7* or ds*Vti1a***

| Acquisition efficiency: No. virus-positive insects/Total no. tested insects | | | |
| --- | --- | --- | --- |
| ds*RNA* | Trial 1 | Trial 2 | Trial 3 |
| ds*GFP* | 46/72 (64%) | 54/65 (83%) | 56/81 (69%) |
| ds*VAMP7* | 44/67 (66%) | 43/58 (74%) | 48/75 (64%) |
| ds*Vti1a* | 32/60 (53%) | 51/67 (76%) | 53/79 (66%) |
