# Supplementary material for: Southern rice black‐streaked dwarf virus hijacks SNARE complex of its insect vector for its effective transmission to rice
Source: Mol Plant Pathol. 2021 Aug 13;22(10):1256–70. doi: 10.1111/mpp.13109 (PMC8435234; doi:10.1111/mpp.13109)
Supplement: Supplementary file 8 — TABLE S3 SRBSDV transmission efficiency by white‐backed planthoppers injected with dsGFP, dsVAMP7, or dsVti1a [file MPP-22-1256-s005.docx]

**Table S3. SRBSDV transmission efficiency by WBPH injected with ds*GFP,* ds*VAMP7* or ds*Vti1a***

| Transmission efficiency: No. virus-positive rice plants/Total no. tested rice plants | | | |
| --- | --- | --- | --- |
| ds*RNA* | Trial 1 | Trial 2 | Trial 3 |
| ds*GFP* | 35/67 (52%) | 47/76 (62%) | 52/75 (69%) |
| ds*VAMP7* | 16/70 (23%) | 19/73 (26%) | 23/79 (29%) |
| ds*Vti1a* | 14/70 (19%) | 19/80 (24%) | 14/82 (17%) |
