# Supplementary material for: Southern rice black‐streaked dwarf virus hijacks SNARE complex of its insect vector for its effective transmission to rice
Source: Mol Plant Pathol. 2021 Aug 13;22(10):1256–70. doi: 10.1111/mpp.13109 (PMC8435234; doi:10.1111/mpp.13109)
Supplement: Supplementary file 9 — TABLE S4 Primers used in this study [file MPP-22-1256-s001.docx]

**Table S4. Primers used in this study**

| **Primer** | **Sequence (5’ - 3’)** |
| --- | --- |
| Y2H-Vamp7-F | ATTAACAAGGCCATTACGGCCATGCTTATCCTATACAGTGTAGTTG |
| Y2H-Vamp7-R | AACTGATTGGCCGAGGCGGCCTCAGCAACTGGACAAAGTCG |
| Y2H-Vti1a-F | ATTAACAAGGCCATTACGGCCATGGCTGCTTTAATTGATGTT |
| Y2H-Vti1a-R | AACTGATTGGCCGAGGCGGCCCCTCGAGAGCGAGTAGCTGATA |
| pFast-SRBSDV P10-F | CGCGGATCCATGGCTGACATAAGACTTGA |
| pFast-SRBSDV P10-R | AACTGCAGTCATCGCATCAGACCCGCAGC |
| pFast-VAMP7-F | CGCGGATCCATGCTTATCCTATACAGTGTA |
| pFast-VAMP7-R | CCCAAGCTTTCAGCAACTGGACAAAGTCGA |
| pFastbac1-Vamp7(cmyc)-R | ACGCGTCGACTCACAGATCCTCTTCAGAGATGAGTTTCTGGCAACTGGACAAAGTCGATCA |
| pFast-Vti1a-F | CGCGGATCCATGGCTGCTTTAATTGATGTT |
| pFast-Vti1a-R | CCCAAGCTTTTATCGAGAGCGAGTAGCTGA |
| dsVAMP7-F | ATTCTCTAGAAGCTTAATACGACTCACTATAGGGATGCTTATCCTATAC |
| dsVAMP7-R | ATTCTCTAGAAGCTTAATACGACTCACTATAGGGCTGGCTGTTAGATTT |
| dsVti1a-F | ATTCTCTAGAAGCTTAATACGACTCACTATAGGGATGGCTGCTTTAATTGATGTTT |
| dsVti1a-R | ATTCTCTAGAAGCTTAATACGACTCACTATAGGGTGCCAGTGCGCTCAACC |
| dsGFP-F | ATTCTCTAGAAGCTTAATACGACTCACTATAGGGGAGCTGTTCACCGGCATCGT |
| dsGFP-R | ATTCTCTAGAAGCTTAATACGACTCACTATAGGGCGATGGGGGTATTCTGCTGG |
| qActin-F | GTATCCTTACCCTTAAGTATCCC |
| qActin-R | TTGAGGGGAGCCTCAGTT |
| qVAMP7-F | ACAGAACAAATCCTGGCGAAAA |
| qVAMP7-R | CAAACTCATCATCCGCAATACA |
| q Vti1a-F | CGCAGAGGGAAACGATACA |
| q Vti1a-R | ACCACAAGGCAGAACACGA |
| qSRBSDV P10-F | CATTCTCCGCTGACGGTTTA |
| qSRBSDV P10-R | TTGAGCAGGAACTTCACGAC |
| pFastbac1-gp64-P8-VSVG-F | ATTAACAAGGCCATTACGGCCTTATGATTGGCACCTATGATGACCGGAT |
| pFastbac1-gp64-P8-VSVG-R | AACTGATTGGCCGAGGCGGCCCCGCAAAGAATAGACACAGCTGTTAAAGGGTTG |
| pFastbac1-gp64-P9-2-VSVG-F | ATTAACAAGGCCATTACGGCCTTATGAACCCACAGTCTTCAGTTAATATTGATACGT |
| pFastbac1-gp64-P9-2-VSVG-R | AACTGATTGGCCGAGGCGGCCCCGTGAAACAAAGTATAATTTACAGTACCTCCATTGAA |
| pFastbac1-gp64-P10-VSVG-F | ATTAACAAGGCCATTACGGCCTTATGGCTGACATAAGACTTGACATAGCGC |
| pFastbac1-gp64-P10-VSVG-R | AACTGATTGGCCGAGGCGGCCCCTCTGGTGACTTTATTTAACACAACCTTTTGACC |
| pFastbac1-gp64-P9-2-1-100aa-VSVG-F | ATTAACAAGGCCATTACGGCCTTATGAACCCACAGTCTTCAGTTAATATTGATACGT |
| pFastbac1-gp64-P9-2-1-100aa-VSVG-R | AACTGATTGGCCGAGGCGGCCCCAAACATTTTAAGCGTTAATTTAAAAATCC |
| pFastbac1-gp64-P9-2-101-209aa-VSVG-F | ATTAACAAGGCCATTACGGCCTTTATCATCTTTTTCGGTGTGTATGTTGT |
| pFastbac1-gp64-P9-2-101-209aa-VSVG-R | AACTGATTGGCCGAGGCGGCCCCGTGAAACAAAGTATAATTTACAGTACCTCC |
